# Characterisation of the Self‐Sufficient Cytochrome P450 CYP116B234 From *Rhodococcus globerulus* and Its Suggested Native Role in 2‐Hydroxyphenylacetic Acid Metabolism

**DOI:** 10.1111/1751-7915.70125

**Published:** 2025-03-08

**Authors:** Simran Kundral, Hannah Beamish, Peter D. Giang, Lauren J. Salisbury, Amanda S. Nouwens, Sunil K. Khare, Paul V. Bernhardt, Jeffrey R. Harmer, Stephen G. Bell, James J. De Voss

**Affiliations:** ^1^ The University of Queensland – Indian Institute of Technology Delhi Research Academy (UQIDRA) Delhi India; ^2^ School of Chemistry and Molecular Biosciences The University of Queensland Brisbane Queensland Australia; ^3^ Department of Chemistry, Enzyme and Microbial Biochemistry Laboratory Indian Institute of Technology Delhi Delhi India; ^4^ Department of Biological Sciences Indian Institute of Science Education and Research Kolkata Kolkata India; ^5^ Centre for Advanced Imaging, Australian Institute for Bioengineering and Nanotechnology The University of Queensland Brisbane Queensland Australia; ^6^ Department of Chemistry The University of Adelaide Adelaide South Australia Australia

**Keywords:** biocatalysis, biodegradation, CYP116, cytochrome P450, oxidation

## Abstract

Cytochromes P450 (P450s) are exceptional biocatalysts that enable the selective oxidation of unactivated C–H bonds using molecular oxygen. Typically, auxiliary redox partner proteins deliver electrons from NAD(P)H to the P450, enabling oxygen activation. However, associating native redox partners with P450s can be challenging, particularly when they are genomically separated. Self‐sufficient P450s, where the P450 heme domain is naturally fused to redox partners, represent a simpler, single‐protein system. Here, we present CYP116B234, a novel self‐sufficient P450 from *Rhodococcus globerulus* , comprising fused heme and phthalate‐family oxygenase reductase (PFOR) domains. The gene encoding CYP116B234 was codon‐optimised for heterologous expression in *E. coli* and subsequently purified to homogeneity. Spectroelectrochemical analysis and electron paramagnetic resonance spectroscopy were performed to determine the redox potentials of the heme and associated Fe–S and FMN cofactors of the PFOR domain. CYP116B234 binds and efficiently oxidises the substituted aromatic compound 2‐hydroxyphenylacetic acid (2‐HPA) to homogentisic acid. Quantitative proteomics revealed the expression of CYP116B234 in *R. globerulus* grown on 2‐HPA, suggesting a role in initiating 2‐HPA degradation. This study presents a new addition to the self‐sufficient CYP116 family and provides evidence for their native function.

## Graphical abstract

A self‐sufficient P450 gene (*CYP116B234*) was identified from *Rhodococcus globerulus* , a soil‐dwelling bacterium isolated from beneath an Eucalypt tree. CYP116B234 consists of heme and reductase domains fused in a single polypeptide. Proteomic analysis suggested the role of CYP116B234 may be to initiate 2‐hydroxyphenylacetic acid catabolism as part of the homogentisate pathway.

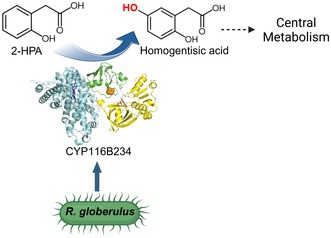

## 1 Introduction

Cytochrome P450 enzymes (P450s) are a superfamily of heme‐thiolate containing monooxygenases that catalyse the regio‐, stereo‐ and chemoselective insertion of an oxygen atom into unactivated C–H bonds (Bernhardt 2006; Guengerich 2019). P450s catalyse many other chemical transformations, such as dealkylation, decarboxylation, demethylation and heteroatom oxidation (Guengerich and Munro 2013; Greule et al. 2018). They are ubiquitous in nature and are involved in the metabolism of numerous physiological substrates such as fatty acids, steroids, vitamins and alkanes (Kelly and Kelly 2013; Urlacher and Girhard 2019).

P450s usually act in conjunction with one or more electron transfer proteins, known as the redox partners (RP), which shuttle electrons from a nicotinamide cofactor NAD(P)H to the catalytic core of the P450 (McLean et al. 2015). This process forms an electron transfer (ET) chain: NAD(P)H → RP → P450 → O_2_, that ultimately results in the heterolytic scission of molecular oxygen bound to the P450 heme. The formation of a reactive iron‐oxo species follows, which performs substrate monooxygenation (Rittle and Green 2010). P450s are currently classified into one of ten classes based on the composition and topology of their redox partners; two of these classes predominate (Hannemann et al. 2007; Finnigan et al. 2020). The most well‐studied is the three‐component Class I system found in prokaryotes and mitochondria that, besides the P450, consists of two redox partners: an iron–sulfur containing ferredoxin (Fdx) and a NAD(P)H‐dependent ferredoxin reductase (FdR). Together, this forms an ET chain: NAD(P)H → FdR → Fdx → P450 required for catalysis. The FdR contains a flavin adenine dinucleotide (FAD) cofactor, and Fdx most often contains a [2Fe–2S] type cluster, but some other types, such as [3Fe–4S] and [4Fe–4S], have also been reported (Conover et al. 1990; Zhang et al. 2014; Child et al. 2018). The best‐studied Class I system is the camphor hydroxylase P450_cam_ (CYP101A1) from *Pseudomonas putida* , which utilises its native RP proteins, putidaredoxin reductase (PdR) and the [2Fe–2S] containing putidaredoxin (Pdx) (Dus et al. 1970). In contrast, two‐component Class II systems are generally found in eukaryotes and consist of a single redox partner, cytochrome P450 reductase (CPR), which contains both an FAD and a flavin mononucleotide (FMN) cofactor (Noble et al. 1999). Bacterial genomes are often replete with electron transfer partner genes, complicating the process of identifying and associating native P450 redox partners unless the P450 and its electron transfer proteins are colocated in the same operon (Smith et al. 2004; Hawkes et al. 2010; Nett et al. 2017; Giang et al. 2023). Bacterial P450s have evolved to be highly specific for their electron transfer proteins, and in most cases, their reconstitution with non‐native partners mostly leads to lower activity (Momoi et al. 2006; Yang et al. 2010; Giang et al. 2024).

A few relatively rare classes of P450 systems exist in bacteria, where the P450 heme domain and redox partners are naturally fused into a single polypeptide chain, thus eliminating the need to identify and separately express electron transfer proteins. These are referred to as the ‘self‐sufficient cytochrome P450 enzymes’ (Roberts et al. 2003) and are mainly classified into two classes (namely class VII and VIII) based on the type and organisation of their redox partners. One of the most widely studied and well‐characterised class VIII self‐sufficient system is P450_BM3_ (CYP102A1), identified from *Bacillus megaterium* . P450_BM3_ is a single polypeptide with the heme domain fused to an NADPH‐cytochrome P450 reductase (CPR) domain. The natural function of P450_BM3_ is proposed to be a long‐chain fatty acid hydroxylase (Cryle et al. 2006); however, over the years, it has been extensively engineered to support the catalytic oxidation of numerous unnatural and structurally different substrates, such as small alkanes and drug metabolites (Meinhold et al. 2005; Thistlethwaite et al. 2021; Fansher et al. 2024). This self‐sufficient nature, complemented by a higher turnover rate (~17,000 min^−1^ for arachidonate hydroxylation) (Davis et al. 1996; Noble et al. 1999) and the capacity to be re‐engineered, has rendered P450_BM3_ as an attractive biocatalyst in producing various high‐value compounds (Sowden et al. 2005).

**FIGURE 1:**
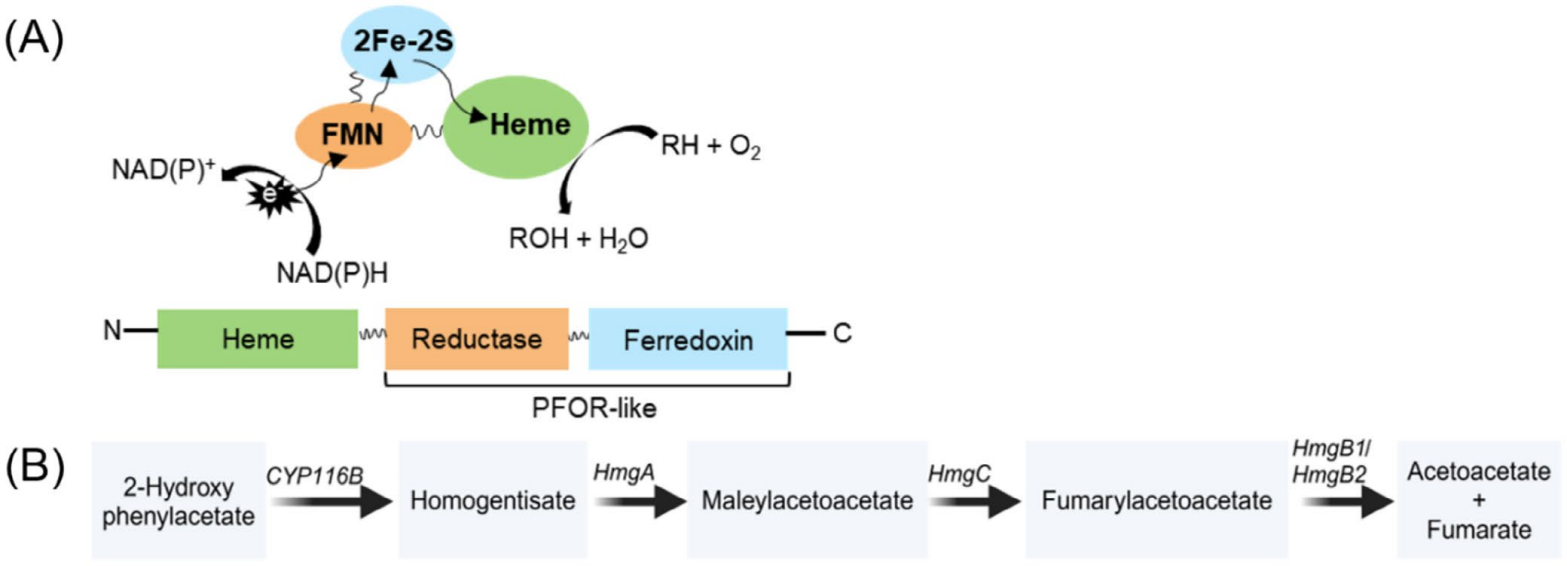
(A) Mechanism of electron transfer in naturally fused class VII CYP116B monooxygenases. The heme domain (green), reductase domain (orange) and ferredoxin domain (blue) are connected with linkers (black). (B) Proposed pathway for the involvement of CYP116B monooxygenase in the catabolism of 2‐HPA via homogentisate in *Cupriavidus pinatubonensis* JMP134 (Donoso et al. 2021). Enzymes involved: HmgA (homogentisate 1,2‐dioxygenase), HmgC (maleylacetoacetate isomerase) and HmgB1/HmgB2 (fumarylacetoacetate hydrolases).

Another type of self‐sufficient system belongs to class VII and includes P450 RhF (CYP116B2) from *Rhodococcus* sp. NCIMB 9784, the first enzyme of the CYP116 family to be characterised (Roberts et al. 2002). The reductase domain, which is fused to the heme domain of P450 RhF, resembles phthalate‐family oxygenase reductase from *Burkholderia cepacia* ; it contains an FMN binding reductase domain and a [2Fe–2S] ferredoxin domain (Batie et al. 1987; De Mot and Parret 2002) (Figure 1A). P450 RhF has been found to catalyse an array of oxidative reactions, including alkane and fatty acid hydroxylation, O‐ and N‐dealkylations, aromatic hydroxylations and the asymmetric sulfoxidation of a range of substituted aromatic compounds (O'Reilly et al. 2013). Notably, P450 RhF has also been reported to hydroxylate a bioactive drug molecule (Klenk et al. 2017). Other self‐sufficient P450s belonging to the CYP116 family were also reported to exhibit a diverse range of reactions and a broad substrate scope, including terpenoid hydroxylation and demethylation of several methoxy compounds (Yin et al. 2014; Porter et al. 2018; Correddu et al. 2021). Despite this catalytic versatility of CYP116 enzymes, it has been difficult to determine their physiological substrate(s). However, the native function of CYP116B enzyme, CYP116B5 from *Acinetobacter radioresistens* S13, has been proposed to be the subterminal and terminal oxidation of long‐ and medium‐chain alkanes, respectively (Minerdi et al. 2015).

In addition, the pollutant‐degrading bacterium *Cupriavidus pinatubonensis* JMP134 contains an *ohpA* gene, which encodes a self‐sufficient cytochrome P450 with 66% amino acid sequence identity to P450 RhF. The native function of OhpA was determined to be involved in the catabolism of 2‐hydroxyphenylacetic acid (2‐HPA) via the homogentisate pathway (Donoso et al. 2021) (Figure 1B). *C. pinatubonensis* is capable of growing on the 2‐, 3‐ and 4‐hydroxy isomers of HPA along with the progenitors of the homogentisate‐ring cleavage pathway, such as tyrosine and phenylalanine. 2‐HPA is a natural phenolic product that can be found in plants of the *Astilbe* genus and derives from the shikimic acid pathway (Kindl 1969). 2‐HPA is believed to be formed through a direct transformation involving the oxidative migration of the side chain of phenylpyruvic acid to yield 2‐HPA; it is then further metabolised to form homogentisic acid. In *Aspergillus nidulans*, the catabolism of phenylacetic acid also proceeds via homogentisate formation, involving two sequential steps, the first of which is ring hydroxylation at the C‐2 position, yielding 2‐HPA (Kluyver and Van Zijp 1951; Rodríguez‐Sáiz et al. 2001). While the enzymes involved in the complete catabolic pathway of such aromatic compounds have only been partially identified, homogentisic acid is thought to be a central intermediate of the pathway in both prokaryotes and eukaryotes. This common intermediate then undergoes a homogentisate‐ring cleavage pathway that involves three enzymes, homogentisate dioxygenase (HmgA), maleylacetoacetate isomerase (HmgC) and fumarylacetoacetate hydrolase (HmgB), to ultimately yield fumarate and acetoacetate for incorporation into the Krebs cycle (Van Den Tweel et al. 1988).

Metabolically versatile bacteria such as those from *Pseudomonas* and *Rhodococcus* are known to contain P450s that catalyse the functionalisation of substrates to yield central metabolites like acetyl‐CoA (Marmulla and Harder 2014). *Rhodococcus* is a genus of aerobic, Gram‐positive bacteria that exhibits a wide range of catabolic capabilities, including the degradation of short‐ and long‐chain alkanes, terpenes and hetero‐ and polycyclic aromatic compounds (Kim et al. 2018). A *Rhodococcus globerulus* strain was isolated as a soil‐dwelling bacterium capable of living on the essential oil of Eucalypt trees. It was determined that two P450s, namely CYP176A1 and CYP108N12, were responsible for initiating the metabolism of terpenes 1,8‐cineole and *p*‐cymene, respectively (Hawkes et al. 2002; Giang et al. 2022). In addition, whole genome sequencing revealed that *R. globerulus* (NCBI GenBank accession no. JACLZG000000000.1) contains approximately 20 other putative P450 genes, with some others already characterised (e.g., CYP108N14 (Giang et al. 2024)), in addition to the ones described above. Interestingly, one of the putative P450 genes identified (now named *CYP116B234*) shared homology to other bacterial self‐sufficient P450s.

This paper reports the heterologous expression, purification and characterisation of CYP116B234, the only self‐sufficient P450 identified within the genome of *R. globerulus* . Given the diverse substrate scope reported for enzymes of the CYP116 family, the putative physiological function of CYP116B234 was explored through both in vitro and in vivo studies. The combination of the self‐sufficient nature and substrate promiscuity of CYP116 enzymes presents them as a promising platform system for mutation studies to enable them to perform biotechnologically useful biotransformations.

## 2 Materials and Methods

### 2.1 General

Bacto‐tryptone was purchased from Thermo Fisher Scientific (Australia). Sodium dithionite, isopropyl‐β‐D‐thiogalactopyranoside (IPTG), nicotinamide adenine dinucleotide phosphate (NADPH), *N*,*O*‐Bis(trimethylsilyl)trifluoroacetamide with trimethylchlorosilane (BSTFA + 1% TMCS), δ‐aminolevulinic acid (ALA) and various substrates such as 3‐(2‐hydroxyphenyl)propionic acid (HPPA) and 2‐hydroxyphenylacetic acid (2‐HPA) were obtained from Sigma‐Aldrich (Australia). All other chemicals used in the study were of analytical grade purity and obtained from commercial vendors.

### 2.2 Phylogenetic Sequence Analysis and Structure Prediction

The protein sequence of self‐sufficient P450 CYP116B234 (GenBank, MCE4265947.1) was identified from the whole genome of *R. globerulus* . Alignment of the amino acid sequences of the catalytic and reductase domains of CYP116B234 with other CYP116 family members was performed using Clustal Omega (Sievers et al. 2011), and phylogenetic analysis was done using the MEGA11 program (Tamura et al. 2021).

The full‐length protein structure of CYP116B234 was predicted by the AlphaFold 3 program (Abramson et al. 2024) and visualised in the PyMOL Molecular Graphics System (Schrödinger LLC 2021).

### 2.3 Heterologous Expression and Purification of CYP116B234

The gene encoding the full‐length sequence of *CYP116B234* from *R. globerulus* was codon‐optimised for *E. coli* , commercially synthesised and cloned into the pET‐28a (+) plasmid (Twist Biosciences, California, United States) to form a pET‐28a (+)/*CYP116B234* construct.

The constructed vector was transformed into *E. coli* BL21 (DE3) competent cells, and single colonies were grown in Lysogeny Broth (LB, 10 mL) supplemented with kanamycin (50 μg mL^−1^) at 37°C (180 rpm) overnight. The following day, Terrific Broth (TB, 500 mL) supplemented with kanamycin (50 μg mL^−1^) was inoculated with seed culture (5 mL) and incubated at 37°C (180 rpm) until the OD_600_ reached 0.6. IPTG (0.4 mM) and ALA (0.25 mM) were added to the culture to induce expression and promote heme biosynthesis, respectively. The culture was further incubated for 20 h at 18°C (180 rpm), after which the cells were harvested by centrifugation (4000 ***g***, 20 min) followed by storage at −80°C until purification.

All purification procedures below were performed at 4°C unless otherwise stated. The harvested cells were resuspended in 100 mM potassium phosphate (KPi) buffer, pH 7.4 (50 mL), supplemented with DL‐dithiothreitol (0.5 mM), phenylmethylsulfonyl fluoride (0.1 mM), and lysozyme (0.1 g L^−1^). Lysis was carried out by sonication on ice (Branson Sonifer 450; 3 × 30 s pulses at 30% output), and the cellular debris was removed by centrifugation (20,000 ***g***, 50 min). The resulting crude lysate was filtered (0.45 μm) and diluted 1:3 with 20 mM KPi buffer, pH 7.4, containing 50 mM NaCl and 10% glycerol (v/v). This solution was then loaded onto a pre‐equilibrated Ni^2+^‐chelating column (HisTrap, Cytiva) using an FPLC system (AKTA FPLC, Cytiva) at 5 mL min^−1^. The column was then washed with 20 mM KPi buffer, pH 7.4, containing 20 mM L‐histidine. CYP116B234 was eluted with a step‐wise gradient of 40–120 mM L‐histidine, and fractions were collected automatically. Fractions with an RZ value (absorption ratio A_418_/A_280_) > 0.6 were combined, concentrated and resuspended in 50 mM Tris–HCl buffer, pH 7.4, before being flash‐frozen in liquid nitrogen for storage at −80°C. P450 concentration was determined by carbon monoxide (CO)‐difference assay, following the established protocol of Guengerich et al. (2009), using *ε*_450nm_ = 91,000 M^−1^ cm^−1^.

### 2.4 FMN Content Quantification

To determine the flavin content, a solution containing CYP116B234 (2 μM) in 50 mM Tris–HCl buffer, pH 7.4, was heated at 90°C for 5 min to release the FMN cofactor, and precipitated protein was removed via centrifugation (12,100 ***g***, 10 min). UV–visible absorbance spectra of the supernatant (i.e., free FMN) were measured. The concentration of free FMN was determined using an extinction coefficient of 12,500 M^−1^ cm^−1^ at 445 nm (Chapman and Reid 1999; Hawkes et al. 2010). The obtained FMN concentration was then compared to the P450 heme concentration determined using a CO‐difference assay.

### 2.5 Iron Content Quantification

Iron content determination was performed by inductively coupled plasma optical emission spectroscopy (ICP‐OES) on an Avio 200 ICP‐OES (Perkin Elmer) (Plasma 8 L min^−1^, Aux 0.2 L min^−1^, neb 0.7 L min^−1^, power 1500 watts, view distance 15) measuring iron emission at 238.204 nm from the axial view of the plasma. The sample flow rate was set to 1 mL min^−1^, with a flush time of 20 s before three replicates were measured with a 15 s delay. The system was washed with ultrapure water, 3 mL min^−1^ for 20 s. Sample iron concentration was determined from the iron standard for atomic absorption spectroscopy (AAS) diluted in ultrapure water (final concentration 0.5–4 mg L^−1^).

### 2.6 Substrate Binding Studies

The binding of the substrate leads to the displacement of a heme‐bound water molecule, changing the spin state of iron from low‐ (∼418 nm) to high‐spin (∼390 nm). To determine substrate binding affinity, difference spectroscopy between substrate‐free CYP116B234 (1 μM) and CYP116B234 (1 μM) titrated with increasing concentrations of substrate was performed until no further spectroscopic changes were observed. The absolute absorbance difference between 390 nm and 420 nm was recorded and plotted against the substrate concentration. The dissociation constant (K_D_) was determined using a typical hyperbolic function unless the substrate exhibited tight binding with K_D_ < 5 μM, in which case a quadratic equation for tight binders was utilised (Morrison 1969) (Equation 1).

The heme iron spin state shift was estimated by comparison with a set of spectra generated from the sum of the appropriate percentages of the spectra of the substrate‐free form (> 95% low spin, Soret peak at 422 nm) and the (*S*)‐limonene bound form (100% high spin, Soret peak at 390 nm) of CYP108N12 (Giang et al. 2022).

(1) $$\Delta A=\Delta A_{\max}\frac{\left(\left[E_{T}\right]+\left[S_{T}\right]+K_{D}\right)-\sqrt{{\left(\left[E_{T}\right]+\left[S_{T}\right]+K_{D}\right)}^{2}-4\left[E_{T}\right]\left[S_{T}\right]}}{2\left[E_{T}\right]}$$

Morrison's quadratic equation for tight binding ligands.

### 2.7 Spectroelectrochemistry

A spectroelectrochemical quartz cell (Pine instruments, 1.7 mm path length) containing (platinum ‘honeycomb’) working electrode and a platinum auxiliary electrode with a separate Ag/AgCl reference electrode (calibrated with quinhydrone, *E*' +267 mV vs normal hydrogen electrode (NHE) at pH 7) was utilised. A solution containing 20 μM CYP116B234 suspended in 50 mM Tris–HCl, pH 7.4, and 10% (v/v) glycerol was prepared, along with another solution containing 20 μM CYP116B234 and 100 μM 2‐HPA (substrate). The following mediator complexes (20 μM) were used: [Co((NMe_3_)_2_)sar]^5+^, [Fe(*trans*–diammac)]^3+^, [Co(AMMEN_3_S_3_sar)]^3+^, [Co(AMMEN_4_S_2_sar)]^3+^, [Co(AMMEN_5_Ssar)]^3+^, [Co(sep)]^3+^, [Co(AMMEsar)]^3+^ and [Co(ClMeClabsar)]^3+^ (Scheme S1). These mediator complexes provide redox buffering across +10 to −450 mV vs NHE and have small extinction coefficients (*ε* < 300 M^−1^ cm^−1^), which avoid contribution to the UV absorption spectra observed (Bernhardt et al. 2006).

Potential dependent UV–visible absorption spectra were measured over 370–800 nm (Ocean Optics USB 2000 fibre optic spectrophotometer with DT‐MINI‐2‐GS mini deuterium/tungsten/halogen UV–Vis–NIR light source) in an anaerobic glove box (O_2_ < 20 ppm). Potentials were set with a potentiostat (BAS100B/W) on constant potential electrolysis mode. Absorption spectra were recorded when absorbance changes ceased. Spectra were measured at 50 mV intervals with decreasing potential followed by interspaced 50 mV intervals with increasing potential, resulting in 25 mV intervals with reversibility. All wavelengths of the potential‐dependent absorbance spectra were analysed and modelled with Reactlab Redox program (Maeder and King 2016) using either a single or double electron transfer model dependent on the cofactor being measured (Equation 2).

(2) $$\text{Absorbance}=\frac{\left(A_{\mathrm{ox}}10^{\frac{n\left(E-E^{0}\right)}{59}}+A_{\mathrm{red}}\right)}{1+10^{\frac{n\left(E-E^{0}\right)}{59}}}$$

Nernst equation and the Beer–Lambert relation for the solution absorbance as a function of solution potential (*E*).

### 2.8 Electron Paramagnetic Resonance

A solution of 35 μM CYP116B234 in 50 mM Tris–HCl (10% (v/v) glycerol, pH 7.4) was prepared with the following organic mediators (40 μM) to provide redox buffering across +29 to −330 mV vs NHE: benzyl viologen, safranine T, anthraquinone sulfonate, 2‐hydroxynaphthaquinone, 2,5‐dihydroxybenzoquinone, and 2,6‐dimethylbenzoquinone. The CYP116B234 sample containing organic mediators was then titrated with 10 mM sodium dithionite (reductant) and 10 mM potassium ferricyanide (oxidant) within an anaerobic glove box (O_2_ < 20 ppm). The reduction potential was measured with a combination of Pt wire and Ag/AgCl electrode (calibrated with quinhydrone, E' + 186 mV vs NHE at pH 7). Upon potential equilibration across the titration, aliquots (50 μL) were transferred to an EPR tube and rapidly frozen in liquid nitrogen after removal from the anaerobic glove box. Continuous‐wave (CW) X‐band (ca. 9.3817 GHz) electron paramagnetic resonance (EPR) spectra were recorded at 17.5 K on a Bruker Elexsys E500 spectrometer equipped with a double‐mode resonator (to correct for *Q*‐value variations) and He cooling (cryogen‐free Bruker system WVGD SYS 5K F70H wRCRC 20). The magnetic field was calibrated with a Gauss meter, and measurements were carried out using a modulation amplitude of 1 mT, a modulation frequency of 100 kHz, and a microwave power of 0.08 mW (34 dB of 200 mW, nonsaturating condition).

### 2.9 NAD(P)H Consumption Assay

The NAD(P)H oxidation rates were determined as previously described (Stok et al. 2016). The reaction mixtures contained purified CYP116B234 (50 nM for NADPH and 1 μM for NADH), catalase (1 μM) and substrate (250 μM) suspended in Tris–HCl buffer (50 mM, pH 7.4). NAD(P)H (150 μM) was added last to initiate the reaction. The rate of NAD(P)H consumption was measured by monitoring the disappearance of absorbance at 340 nm over 1 min, using an extinction coefficient of NAD(P)H, *ε*_340nm_ = 6220 M^−1^ cm^−1^.

### 2.10 In Vitro CYP116B234 Turnovers

A solution of Tris–HCl buffer (50 mM, pH 7.4) containing purified CYP116B234 (1 μM), catalase (1 μM), substrate (100 μM) and NADPH (200 μM) was left to stir for 30 min at room temperature (~25°C). An appropriate volume of HCl (1 M) was then added to the reaction mix to reach pH 0–2 in order to protonate the organic acid. The mixture was subsequently extracted with ethyl acetate (3 × 1 mL), dried over magnesium sulfate (MgSO_4_) and concentrated *in vacuo*. The dried sample was then dissolved in ethyl acetate (20 μL) and an equal volume of BSTFA‐TMCS was added. The sample was heated at 85°C for 15 min before being analysed by GC–MS (Shimadzu GC QP2030, Zebron ZB‐5MS column) with the following temperature program: 50°C for 5 min, a 10°C min^−1^ increase until 300°C was reached and held for 10 min.

For coupling efficiency measurements, the reaction mixtures contained CYP116B234 (1 μM), catalase (1 μM), substrate (250 μM) and NADPH (200 μM) in Tris–HCl buffer (50 mM, pH 7.4). The reactions were incubated at room temperature (~25°C) for 30 min. At the end of the reaction, an internal standard (100 μM) was added prior to acidification and extraction. Product quantification was performed using a substrate standard curve (50–250 μM) with the internal standard (100 μM).

### 2.11 Whole‐Cell Biotransformation

The pET28a (+) plasmid carrying the codon‐optimised *CYP116B234* gene was expressed in *E. coli* BL21(DE3) cells following the same protocol described above for the heterologous expression. Simultaneously, the same procedure was carried out on *E. coli* BL21(DE3) cells transformed with an empty pET28a (+) vector as a negative control. P450 expression was confirmed by a CO difference assay on the crude cell lysate. The cell pellet from the expression culture (500 mL) was resuspended in M9 minimal media (100 mL) (Sambrook 2001) with 2‐HPA (2 mM) and incubated at 25°C, 180 rpm for 16 h, and the cell‐free extract was then obtained via centrifugation (5000 ***g***, 20 min). The cell‐free extracts were acidified (pH 0–2) using HCl to protonate the organic acid. Products were then extracted with ethyl acetate (3 × 50 mL), dried over magnesium sulfate and concentrated (50 μL) *in vacuo*. Concentrated samples were derivatised using BSTFA‐TMCS and analysed using GC–MS.

### 2.12 Determination of CYP116B234 Biological Function and Proteome Analysis

*R. globerulus* was cultured in enrichment media (Peterson and Lu 1991) containing either phenylalanine, phenylacetic acid, or 2‐HPA (5 mM) as the single source of carbon and energy at 26°C for 20 h (120 rpm). If growth was observed, cells were subcultured at a 1/10 (final volume 10 mL) dilution into fresh media with the same substrate concentration (5 mM) until an OD_600_ of 0.6 was reached. This dilution and subculturing process was repeated five times before scaling up to large‐volume cultures (4 × 500 mL). The cells were harvested at an OD_600_ of 1 by centrifugation at (5000 ***g***, 20 min), and stored at −80°C until further purification at 4°C. The resulting cell pellet was resuspended in Buffer A (50 mM Tris–HCl, pH 7.4) supplemented with 0.1 mM PMSF, 0.5 mM DTT and 0.1 g L^−1^ lysozyme. The resuspended cells were lysed by sonication (3 × 30 s at 30% output) and debris was removed by centrifugation (20,000 ***g***, 50 min). A quantitative analysis method, Sequential Window Acquisition of All Theoretical Mass Spectra (SWATH‐MS), was employed for a relative protein content analysis in the crude cell lysate. To prepare the cell lysate for SWATH‐MS analysis, filter‐aided sample preparation (FASP) methodology was carried out following the procedure of Wiśniewski et al. (2009).

Simultaneously, the crude lysate was diluted 1/3 in Buffer A and loaded onto a pre‐equilibrated MonoQ anion exchange column (HiPrep Q HP 16/10, Cytiva) using an FPLC system (AKTA FPLC, Cytiva) at 2 mL min^−1^. CYP116B234 was eluted using a linear salt gradient (0–800 mM KCl), and 3 mL fractions were collected automatically. The presence of P450 in the eluted fractions was confirmed via a CO‐difference assay.

Protein mass spectrometric analysis was subsequently performed after in‐solution tryptic digestion. Briefly, purified protein fractions were treated with DTT (5 mM) and acrylamide (25 mM) to reduce the disulphide bonds and alkylate the free cysteine residues, respectively. Samples were then diluted with ammonium bicarbonate, followed by trypsin digestion (10 ng μL^−1^) at 37°C overnight. The following day, peptides were extracted and purified using a ZipTip C18 cartridge.

Alternatively, for further analysis, the purified protein fractions were subjected to SDS‐PAGE (NuPAGE 4%–12%) and the band of interest was excised from the gel for in‐gel tryptic digestion. Both in‐solution and in‐gel digested peptides were processed for mass spectrometry, as detailed in the Supporting Information.

## 3 Results and Discussion

### 3.1 Phylogenetic Analysis, Sequence Alignment and Structure Prediction of CYP116B234

**FIGURE 2:**
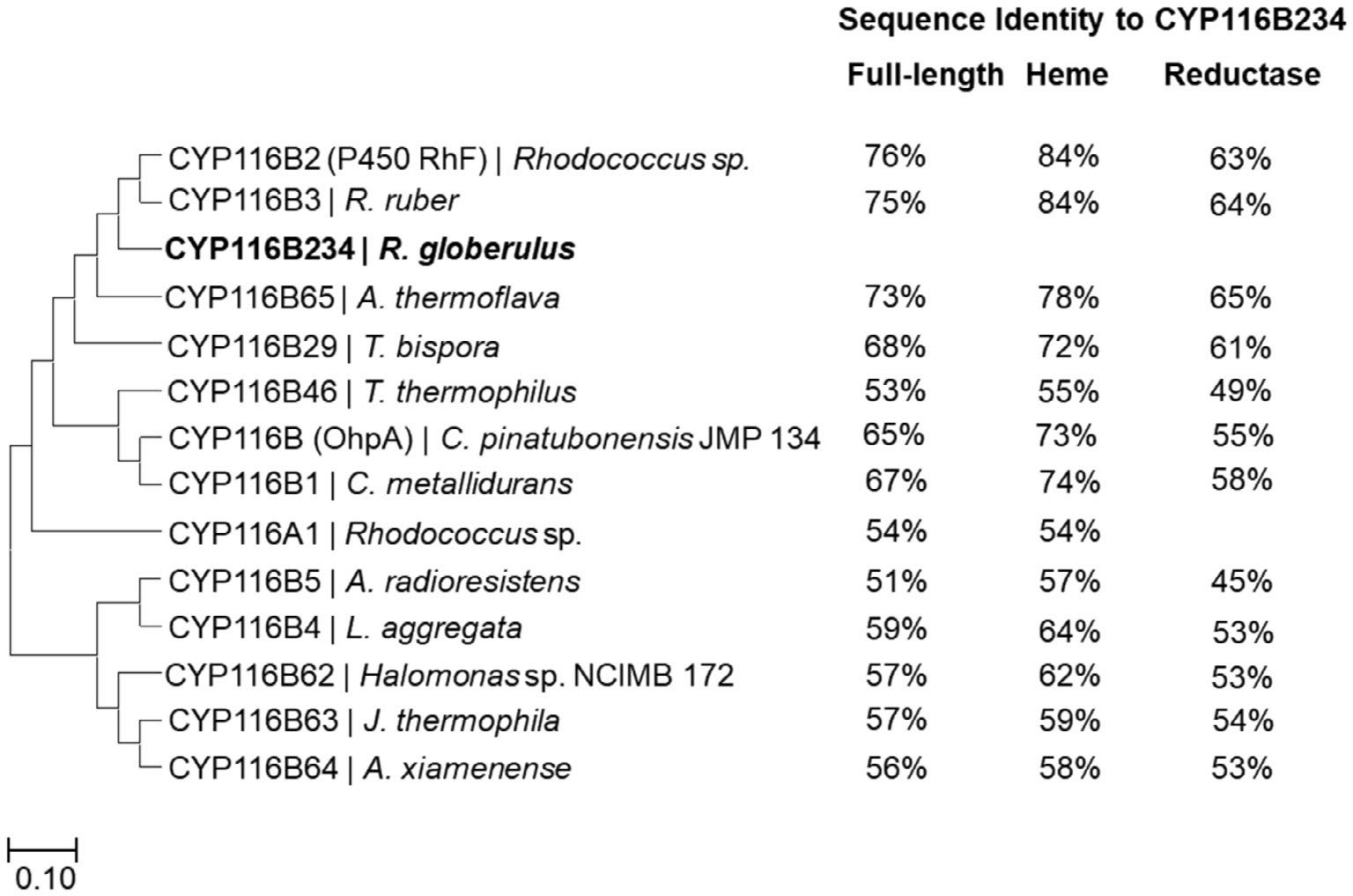
Phylogenetic tree of characterised CYP116 family enzymes. Amino acid sequences were aligned using the Clustal Omega multialignment tool, and the tree was built using the neighbour‐joining method (MEGA11).

The genome sequencing of this *R. globerulus* strain (NCBI GenBank accession no. JACLZG000000000.1) revealed the presence of a single class VII self‐sufficient P450, *CYP116B234*. To compare CYP116B234 with other CYP116 family enzymes, full‐length protein sequences were aligned using Clustal Omega (Figure S1) and a phylogenetic tree was constructed using the neighbour‐joining method (Figure 2). The heme domain of CYP116B234 shared the highest sequence identity (> 80%) with both the well‐characterised CYP116B2 (P450 RhF) and CYP116B3, indicating that the putative P450 enzyme belongs to the CYP116B subfamily. The reductase domain of CYP116B234 shared the highest identity (> 60%) with the PFOR domain of CYP116B65, CYP116B2, CYP116B3 and CYP116B29. While the reductase domain of CYP116B234 shared relatively less homology compared to its heme domain and those of other CYP116B enzymes, it was still significant and suggested that CYP116B234 contains a phthalate family oxygenase reductase (PFOR) type domain that consists of a [2Fe–2S] cluster containing ferredoxin domain and an FMN‐containing NAD(P)H reductase domain.

**FIGURE 3:**
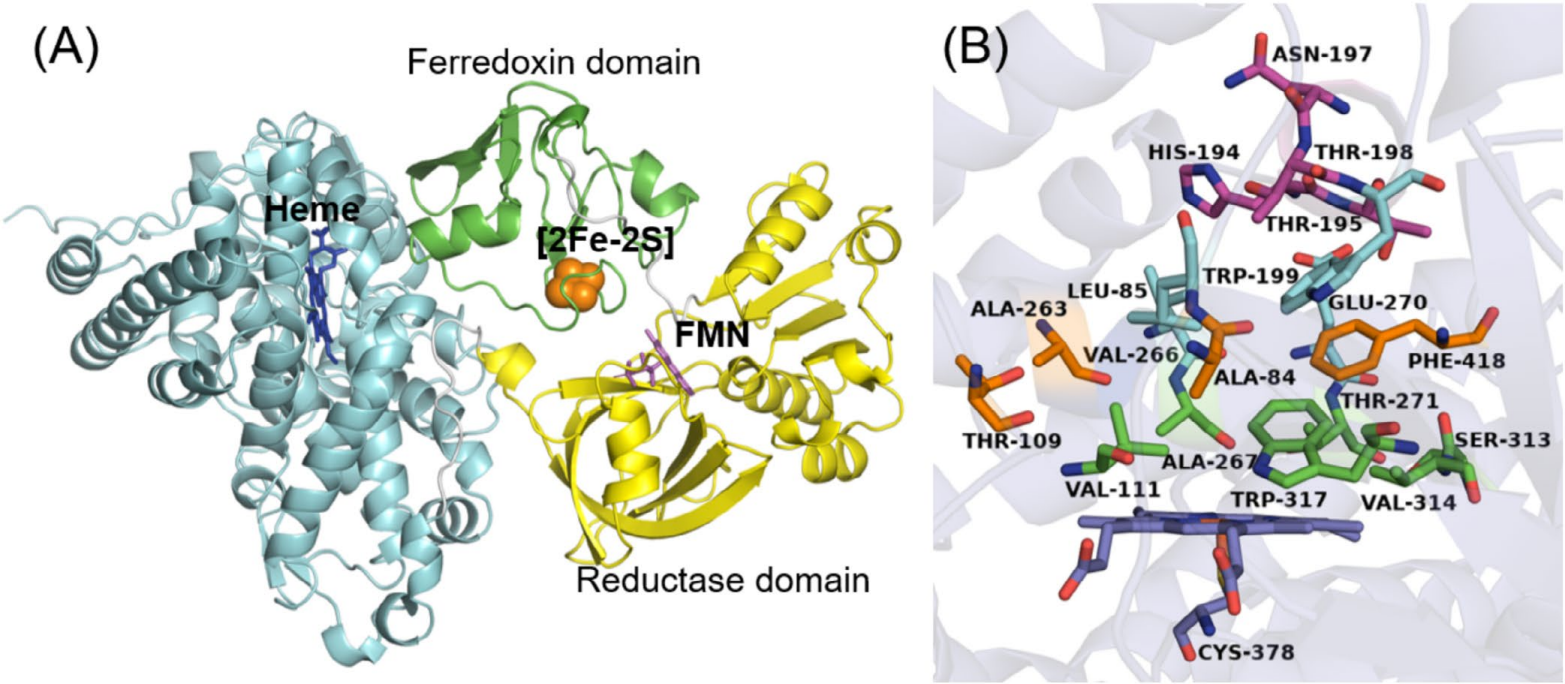
Predicted structure of CYP116B234 using AlphaFold 3 and visualised in PyMol (A) The full‐length structure is presented as a cartoon showing heme, [2Fe–2S] and FMN coloured in blue, orange and magenta, respectively. (B) Focused image showing the four tiers of active‐site residues: Tier 1 (green), tier 2 (orange), tier 3 (cyan) and tier 4 (magenta).

The high protein sequence identity of CYP116B234 with other CYP116B enzymes prompted further analysis of the catalytic pocket residues to evaluate their influence on substrate profiles. The first crystal structure of a Class VII holoprotein (CYP116B46) revealed that its active site is embedded within the substrate access channel, surrounded by 18 residues arranged in four distinct tiers above the heme cofactor (Tavanti et al. 2018a). To explore the structural features of CYP116B234, the 3D protein structure was predicted using AlphaFold 3, which enabled the similar visualisation of the four tiers of residues lining the catalytic pocket (Figure 3). Most of the active site residues are conserved between CYP116B234 and CYP116B46, with a few notable differences. For instance, Ser313 in Tier 1 replaces Pro320 of CYP116B46, Ala84 replaces a homologous residue Val91 in Tier 2, Trp199 replaces Phe206 in Tier 3, and Thr198 replaces Ala205 in Tier 4.

Protein sequence alignment between OhpA (CYP116B2 homologue from *C. pinatubonensis* JMP134) and CYP116B234 revealed a 65% sequence identity, with all 18 active site residues fully conserved. Likewise, CYP116B5 from the alkane‐degrading *A. radioresistens* S13 shows complete conservation of these active site residues with CYP116B234. Sequence alignment studies further revealed that these residues are largely conserved across the CYP116B family, with some conservative substitutions. For instance, Val314 in Tier 1 is often substituted by the homologous residue isoleucine (70% conserved) (Donoso et al. 2021). In enzymes with a buried active site, such as cytochrome P450s, both the residues within the active site and those lining the substrate access channel play crucial roles in determining substrate specificity. Although the high similarity in these residues among CYP116B enzymes provides valuable insights, it is insufficient to determine substrate preference. Thus, binding and catalytic activity assays remain essential to establish the substrate profiles for these enzymes.

The crystal structure of CYP116B46 also highlights the involvement of key residues (Arg718, Glu723, Ser726, Glu729 and Arg388) in electron shuttling from [2Fe–2S] to the heme. Alignment of CYP116B234 and CYP116B46 revealed that the residues Arg718, Ser726 and Glu729 are conserved in CYP116B234, while Glu723 and Arg388 are replaced by homologous residues Asp715 and Lys381, respectively. These substitutions suggest that CYP116B234 likely follows a similar electron transfer mechanism to that of CYP116B46 (Figure S2).

### 3.2 Expression, Purification and Spectroscopic Characterisation of CYP116B234

The pET28a (+) plasmid carrying the codon‐optimised *CYP116B234* produced the encoded enzyme recombinantly in *E. coli* BL21(DE3) cells at approximately 440 nmol L^−1^ of culture. The intrinsic N‐terminal polyhistidine tag encoded by the plasmid was utilised to purify CYP116B234 using Ni‐NTA affinity chromatography. Following the single‐step purification, CYP116B234 was isolated with an R/Z of approximately 0.74. The presence of a single band on SDS‐PAGE at the expected mass (85 kDa) indicated the high purity of the purified protein (Figure S3).

**FIGURE 4:**
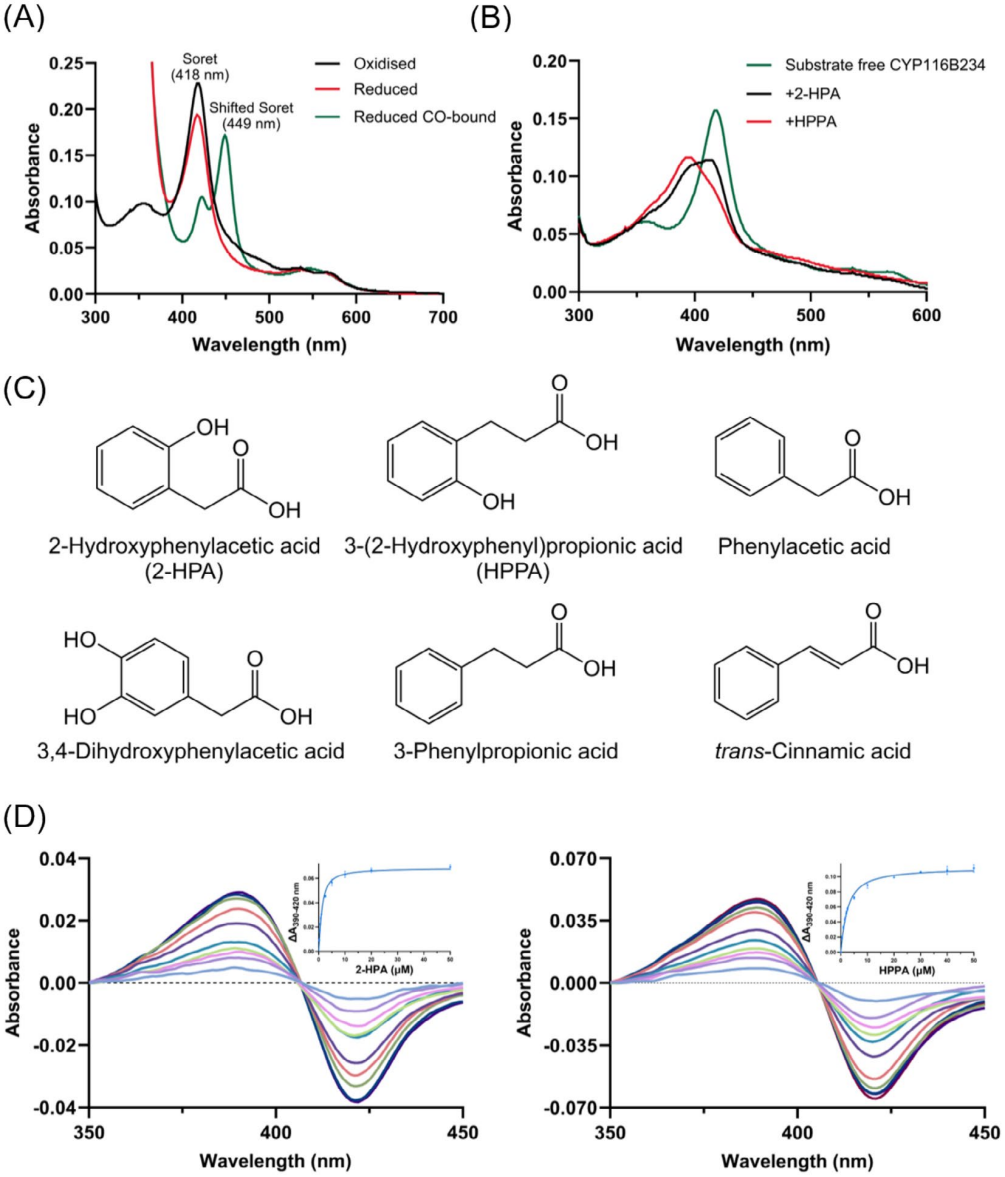
(A) UV–visible spectra (300–700 nm) of purified CYP116B234 in its oxidised (black), reduced (red) and reduced CO‐bound forms (green). (B) UV–visible spectra (300–600 nm) of CYP116B234 (1 μM; green) in the presence of substrates 2‐HPA (100 μM; black) and HPPA (100 μM; red). (C) Structures of compounds tested for binding to CYP116B234. (D) UV–visible difference spectra (350–450 nm) and inset corresponding binding curve obtained by titration of CYP116B234 with increasing concentrations (0.2–50 μM) of substrates 2‐HPA and HPPA.

UV–visible spectroscopy was used to characterise the purified protein. As shown in Figure 4A, the oxidised ferric form of CYP116B234 exhibited the characteristic heme Soret peak at 418 nm along with α and β bands at 566 and 536 nm, respectively. Upon chemical reduction (sodium dithionite), the heme Soret reduced in intensity. The addition of carbon monoxide to reduced CYP116B234 showed the expected shift in the Soret maximum from 418 nm to 449 nm, indicative of a properly folded P450.

To quantitate the incorporation of the flavin cofactor during the expression of CYP116B234, the purified protein was heat denatured at 90°C to release the bound FMN, and the yellow‐coloured supernatant was then analysed via UV–visible absorbance spectroscopy. The concentration of free FMN (*ε*_445nm_ = 12,500 M^−1^ cm^−1^) in comparison with the heme was determined to be 0.75 per heme, suggesting that 75% of CYP116B234 contained both the heme and FMN cofactors. This was then compared to CYP116B234 purified after strong MonoQ anion exchange column following the procedure of Giang et al. (2022), during which FMN was seen to elute separately from the P450 heme, and only 22% of the starting protein was eluted with both the heme and FMN domains intact (data not shown).

In addition to FMN, the self‐sufficient CYP116B234 also contains a [2Fe–2S] cluster, and ICP‐OES was performed to measure the iron content in purified CYP116B234. The iron content of purified CYP116B234 was compared to that determined for CYP199A4 (Bell et al. 2009), given that the latter contains a single iron rather than the three expected in CYP116B234 (one from the P450, two from the ferredoxin). A sample of 5.7 μM CYP116B234 was found to contain 0.921 ± 0.008 mg L^−1^ iron (16.5 μM), while CYP199A4 of the same concentration contained 0.338 ± 0.004 mg L^−1^ (6.05 μM). Both compare well with the expected value of 3 Fe for CYP116B234 and 1 Fe for CYP199A4, indicating full incorporation of Fe into the purified CYP116B234 enzyme.

### 3.3 Substrate Binding Analysis

Substrate binding to a P450 can lead to the displacement of a heme‐bound water molecule, changing the heme iron from low to high spin. This is observable by UV–visible spectroscopy, with the heme Soret peak at ~418 nm (low spin) shifting towards ~390 nm (high spin). Due to the wide substrate range reported for the CYP116 family, a collection of substrates composed of fatty acids, steroids, terpenes and aromatic compounds was tested for the ability of each to bind to CYP116B234.

Out of all the substrates tested (Figure 4C), two substituted aromatics, namely 2‐HPA and HPPA, exhibited the highest heme spin state change (66% ± 1%, 90% ± 1% respectively). For both substrates, not all of the heme iron changed to a high‐spin state in the presence of excess substrate (Figure 4B). Both substrates bound tightly to CYP116B234 with K_D_ values of 0.7 ± 0.1 μM for 2‐HPA and 1.9 ± 0.1 μM for HPPA (Figure 4D).

Additionally, substrates structurally similar to the two above‐mentioned compounds were also tested, including phenylacetic acid, 3,4‐dihydroxyphenylacetic acid, 3‐phenylpropionic acid and *trans*‐cinnamic acid. However, all of these substrates induced a much smaller spin state shift (2% ± 1%, 5% ± 3%, 8% ± 5%, 8% ± 4%) than either of the two tight binders, 2‐HPA and HPPA (Table S1). Therefore, substrate binding to CYP116B234 seems to require the presence of a hydroxyl group at the *o*‐position of the ring and not at either the *m*‐ or *p*‐positions. Other substrates, including fatty acid, terpenoids and steroids, also bound poorly and induced small changes in the UV–Vis absorbance spectrum (< 10% high spin, Table S1).

### 3.4 Redox Characterisation

**FIGURE 5:**
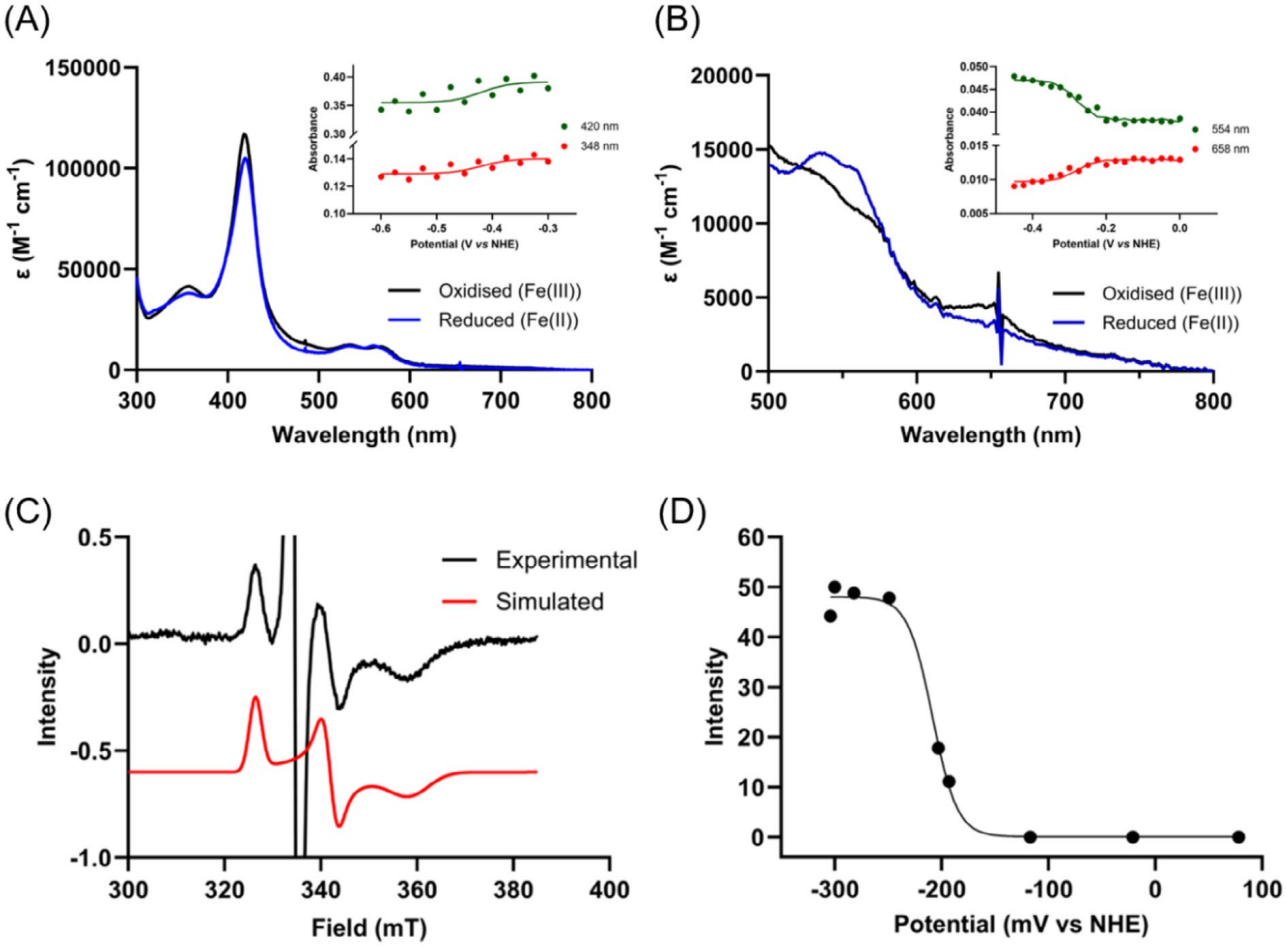
(A) UV–visible spectra (300–800 nm) of substrate‐free CYP116B234 (20 μM) showing fully oxidised and fully reduced states. Inset selected single wavelength absorption values due to applied potential. (B) UV–visible spectra (500–800 nm) of spectrochemical titration of substrate‐bound (100 μM 2‐HPA) CYP116B234 (20 μM) showing fully oxidised and fully reduced states. Inset selected single wavelength absorption values due to applied potential. The calculated redox potential of substrate‐bound CYP116B234 was −270 mV vs NHE. (C) X‐band (9.3817 GHz) CW EPR spectra recorded at 17.5 K of CYP116B234 (35 μM, 20 K) at −249 mV (vs NHE). The large signal at 335 mT is from the benzyl viologen radical cation. The black line present experimental data and red line is the simulated Fe–S cluster *g* = [2.05, 1.96, 1.87]. (D) CYP116B234 [2Fe–2S] signal intensity obtained by the fitted simulations as a function of potential vs NHE. The data were fit to a single electron process. CYP116B234 [2Fe–2S] *E*_m_ = −212 mV ± 20 vs NHE.

As CYP116B234 contains three distinct redox centres (namely a heme, a [2Fe–2S] cluster, and an FMN), the redox potentials of each of these centres were explored through a combination of spectroelectrochemistry and EPR. Spectroelectrochemical experiments were performed to determine the redox potential of the heme and FMN cofactors. Given the relatively large extinction coefficient of P450 heme groups (*ε*_418nm_ = ~90,000–100,000 M^−1^ cm^−1^), changes in its spectral properties as a result of the heme iron's reduction or oxidation were hypothesised to be distinct and obvious. For example, the reduction of CYP176A1 (P450_cin_, also from *R. globerulus* ) resulted in the spectral shift of the heme Soret absorbance from 415 nm to 411 nm (Hawkes et al. 2002). However, similar to the chemical reduction of CYP116B234 by sodium dithionite (Figure 4A), the electrochemical reduction of CYP116B234 resulted in only a decrease in absorbance of the heme Soret and no significant spectral changes were detected in the α‐β region upon reduction (Figure 5A). This behaviour has been reported previously for CYP124A1 commonly found in the *Mycobacterium* genus, where upon the reduction of the substrate‐free P450, only a decrease in absorbance of the heme Soret at the same wavelength (420 nm) accompanied by small spectral changes in the α‐β region was observed (Mohamed et al. 2023). Based on the minimal spectral changes of the heme Soret, the redox potential of low‐spin substrate‐free CYP116B234 was tentatively assigned as −430 mV vs NHE.

In contrast, the electrochemical reduction of substrate‐bound (100 μM 2‐HPA) CYP116B234 resulted in fast and reversible potential‐dependent spectroscopic changes (Figure 5B). Analysis of the heme Soret was more difficult due to 2‐HPA not completely shifting CYP116B234 to the high‐spin state, resulting in the presence of both high‐and low‐spin heme iron (Figure S4). Spectral changes at longer wavelengths (554 nm and 658 nm) are more obvious and also are unaffected by absorbance due to either the [Fe–S] or FMN cofactors. The heme redox potential of CYP116B234 with 2‐HPA bound was calculated to be −270 mV vs NHE.

The redox potential of the FMN chromophore of CYP116B234 was also successfully determined through UV–Vis spectroelectrochemistry. When attempted with substrate‐bound CYP116B234, it was found that the redox potential of the heme iron and FMN domain overlapped significantly and, therefore, could not be determined. However, the spectroelectrochemical reduction of the substrate‐free form of the enzyme showed reversible spectral changes in the range 450–500 nm (typical of the FMN chromophore) upon electrochemical reduction and oxidation. The resultant spectral changes as a function of potential are shown in Figure S5 with the calculated redox potential of −240 mV vs NHE derived from the analysis of the entire potential dependent spectral data.

Despite successfully determining the redox potential of the heme and FMN domains in CYP116B234 through spectroelectrochemistry, the redox potential of the [2Fe–2S] cluster was unable to be unambiguously determined due to the competing heme and FMN chromophores. Therefore, EPR spectroscopy was employed to measure the redox potential of the [Fe–S] cluster. Upon reduction of substrate‐bound CYP116B234, the [2Fe–2S] signal intensity increased at 326 and 340 mT, respectively (Figure 5C). As a result, g‐values of 2.05 (g_1_), 1.96 (g_2_) and 1.87 (g_3_) were obtained, consistent with the presence of a [2Fe–2S] cluster. A redox potential of −212 mV ± 20 vs NHE was subsequently determined by fitting the relationship between the intensity of the [2Fe–2S] EPR signal and potential to a single electron process (Figure 5D). We observed that the redox potentials of the substrate‐free and substrate‐bound heme, measured by EPR, were significantly higher than those determined through spectroelectrochemistry. This discrepancy may arise from differences in experimental conditions (e.g., temperature, pH and mediators) between the two techniques, which can influence the redox behaviour of the heme. Further investigation is necessary to fully understand and account for this variation.

The potentials obtained for the reductase domain of CYP116B234 are highly comparable to those reported for the intact P450 RhF, where the two‐electron reduction potential of FMN was −243 ± 15 mV vs NHE (Roberts et al. 2003) and the [2Fe–2S] cluster potential, determined from the isolated FeS domain, was −214 mV vs NHE (Hunter et al. 2005). The heme redox potential of intact P450 RhF in the absence of substrate was determined to be −423 ± 10 mV vs NHE. In our study, the identification of 2‐HPA as a substrate for CYP116B234 enabled the first determination of the heme redox potential in the presence of a substrate (−270 mV vs NHE).

### 3.5 NAD(P)H Consumption Assay

Steady state kinetics were performed to determine the rate of substrate‐stimulated oxidation of NAD(P)H, indicated by a decrease in absorbance at 340 nm. The observed rate of NADPH oxidation in the presence of 2‐HPA (423 ± 10 μM (μM CYP)^−1^ min^−1^) was significantly higher than for HPPA (143 ± 1 μM (μM CYP)^−1^ min^−1^) (Table 1). Notably, the corrected values of oxidation rates for full FMN incorporation are also provided in Table 1. Interestingly, despite HPPA inducing a greater spin shift with CYP116B234, 2‐HPA both binds approximately 2.7‐fold more tightly to the enzyme and has a faster NADPH oxidation rate.

**TABLE 1:** Comparative analysis of binding affinity, NADPH consumption kinetics, and coupling efficiency of CYP116B234 with substrates 2‐HPA and HPPA. NADPH consumption rates were normalised by subtracting the background rate due to the reductase domain and further corrected for complete FMN incorporation. Coupling was calculated as a percentage of substrate depleted/NADPH consumed.

Substrate	Spin state shift (%)	K_D_ (μM)	NADPH consumption rate (μM (μM CYP116B234)^−1^ min^−1^)	NADPH consumption rate (corrected for complete FMN incorporation)	Coupling (%)
2‐hydroxyphenylacetic acid (2‐HPA)	66 ± 1	0.7 ± 0.1	423 ± 10	564 ± 10	86
3‐(2‐hydroxyphenyl) propionic acid (HPPA)	90 ± 1	1.9 ± 0.1	143 ± 1	190 ± 1	4

Furthermore, the oxidation rate of NADPH with 2‐HPA was significantly higher than that of NADH (125 ± 1 μM (μM CYP)^−1^ min^−1^). The preference for the NADPH cofactor by CYP116B234 is in agreement with other self‐sufficient CYP116B enzymes belonging to Class VII and also with CYP102 enzymes belonging to Class VIII, all of which prefer NADPH over NADH (Maddigan and Bell 2017; Tavanti et al. 2018b). The Michaelis–Menten constant (K_M_) measurements further confirmed this preference, with NADPH exhibiting a significantly lower K_M_ (0.5 ± 0.4 μM) compared to NADH (240 ± 20 μM) (data not shown). These findings highlight the enzyme's preference for 2‐HPA as a substrate and NADPH as a cofactor.

### 3.6 In Vitro Catalytic Turnovers

**FIGURE 6:**
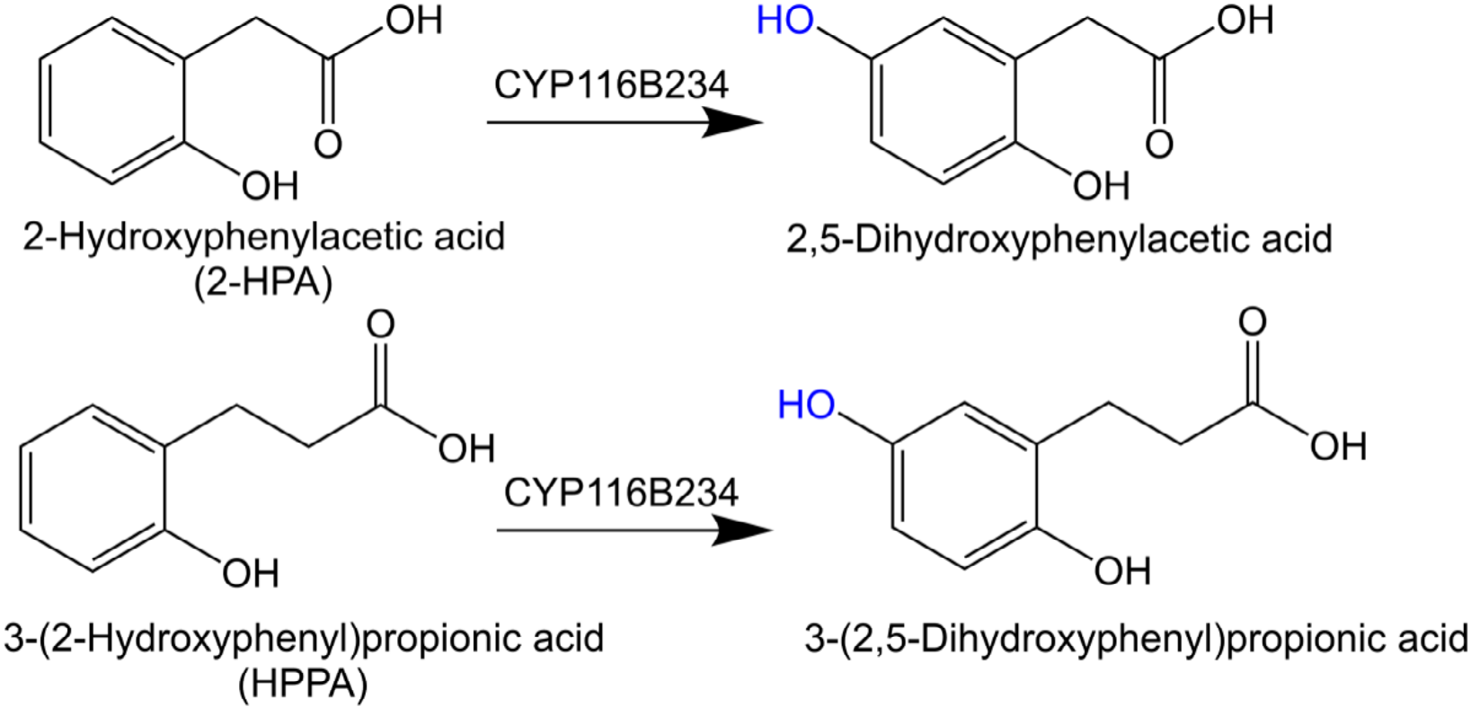
Products of in vitro catalytic turnover of 2‐HPA and HPPA in the presence of NADPH.

**FIGURE 7:**
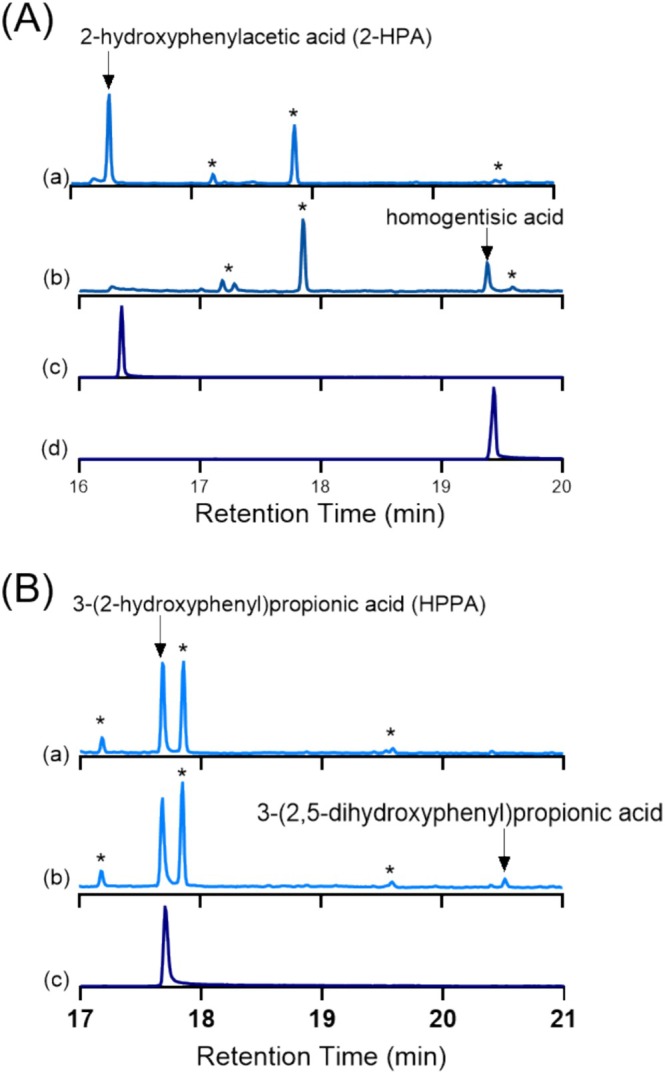
GC–MS traces of in vitro turnovers by CYP116B234 of substrates (A) 2‐HPA (a) negative control, (b) 2‐HPA turnover, (c) 2‐HPA commercial standard with a retention time of 16.3 min (m/z 296), and (d) homogentisic acid standard with a retention time of 19.4 min (m/z 384). (B) HPPA (a) negative control, (b) turnover of HPPA giving product 3‐(2,5‐dihydroxyphenyl)propionic acid with a retention time of 20.5 min (m/z 398), and (c) HPPA standard with a retention time of 17.7 min (m/z 310). Other peaks marked as stars (*) in traces (a) and (b) are various organosilicon contaminants with mass fragmentation patterns unrelated to the substrate or product. All samples were derivatised with BSTFA‐TMCS before GC–MS analysis.

In vitro turnovers with CYP116B234 in the presence of NADPH revealed that CYP116B234 catalysed the hydroxylation of 2‐HPA to 2,5‐dihydroxyphenylacetic acid (homogentisic acid) and HPPA to 3‐(2,5‐dihydroxyphenyl)propionic acid (Figure 6). The identities of the products were confirmed by GC–MS analysis through comparison with authentic commercial standards, which exhibited identical retention times and mass fragmentation patterns, or in the case of HPPA, from the unique mass fragmentation profile which matched that reported by Heindl et al. (1985) (Figures S6 and S7). When the reaction was conducted in the presence of a stoichiometric amount of NADPH relative to the substrate, complete consumption of 2‐HPA was observed (Figure 7A); however, complete conversion of HPPA was not achieved under similar conditions (Figure 7B).

The coupling efficiency was also calculated to determine the proportion of the electrons consumed from NADPH that resulted in organic product formation. Product quantification is usually achieved by correlating the peak area of the product to that of the internal standard used. However, the quantification of the homogentisic acid peak was unreliable due to its tendency to be further converted to another compound, which was not detected using GC–MS. The same phenomenon was observed by Donoso et al. (2021) where the homogentisic acid produced was reported to undergo auto‐oxidation and polymerisation to an extracellular pigment called pyomelanin. CYP116B234 likely generated homogentisic acid, which then auto‐oxidised to pyomelanin. Therefore, for the calculation of catalytic efficiency, we utilised substrate depletion to determine the oxidation efficiency. With 2‐HPA, CYP116B234 was tightly coupled and utilised 86% of the consumed NADPH for product formation. Comparatively, only 4% of the NADPH consumed in the oxidation of HPPA resulted in product formation. Typically, the electrons consumed in these reactions that do not go to organic product formation result in hydrogen peroxide or water production. The vast difference in coupling efficiency between both substrates indicates that 2‐HPA is the preferred substrate for CYP116B234.

### 3.7 In Vivo Whole‐Cell Oxidations

Whole‐cell oxidations using an *E. coli* whole‐cell‐based system were performed to further validate the oxidative capabilities of CYP116B234 and identify any potential metabolites generated. After the successful expression of CYP116B234 holoenzyme, cells were harvested and resuspended in M9 minimal media with a single addition of the substrate 2‐HPA (2 mM). GC–MS analysis revealed the complete oxidation of 2‐HPA and the generation of a single metabolite identified as homogentisic acid by comparison with an authentic commercial standard (Figures S8 and S9). It was observed that the size of the peak of the product was significantly smaller than the substrate present in the negative control, clearly indicating again that GC–MS analysis does not allow the quantification of the product homogentisic acid. This discrepancy further suggested that the homogentisic acid may be metabolised to another compound, possibly pyomelanin. Such a polymer would not be able to be detected by GC–MS due to its size, explaining the lack of visibility of the product peak from the complete conversion of the substrate. Furthermore, following the incubation of *E. coli* cells expressing CYP116B234 with 2‐HPA, the culture turned brown, which is the expected colour of pyomelanin, while the negative control did not undergo a change of colour (Figure S10).

### 3.8 Determination of CYP116B234 Biological Function and Proteome Analysis

In order to investigate the native function and involvement of CYP116B234, *R. globerulus* was cultured on enrichment media containing a single carbon source, which was either 2‐HPA, phenylalanine, or phenylacetic acid; the latter two compounds are potential precursors of 2‐HPA. *R. globerulus* was found to grow on 2‐HPA and phenylalanine as sole carbon sources; however, it failed to grow on phenylacetic acid.

Proteomic analysis (SWATH‐MS) of cell lysates from *R. globerulus* grown on 2‐HPA or phenylalanine revealed distinct protein expression profiles. In phenylalanine‐grown cells, no P450 enzymes were detected. Instead, enzymes involved in phenylalanine catabolism via tyrosine, such as phenylalanine 4‐hydroxylase and carbinolamine dehydratase, were identified (Figure S11, Table S2). In contrast, CYP116B234 was detected in 2‐HPA‐grown cells, albeit with low amino acid sequence coverage (8%), suggesting low abundance (Table S3). Interestingly, enzymes from the homogentisate central pathway, including homogentisate 1,2‐dioxygenase (HmgA) and fumarylacetoacetate hydrolase (HmgB), were present in both phenylalanine‐and 2‐HPA‐grown cells. This suggests that both phenylalanine and 2‐HPA are channelled through the homogentisate pathway in *R. globerulus* .

To further confirm the involvement of CYP116B234 in the catabolism of 2‐HPA, cell lysate from *R. globerulus* grown on 2‐HPA was fractionated using anion exchange chromatography (MonoQ). Fractions displaying an absorbance peak at 418 nm, indicative of cytochrome P450 enzymes, were collected. A UV–visible CO difference assay of these fractions indicated the presence of P450 at approximately 1.2 nmol L^−1^, reflecting low expression levels of P450; these fractions were subsequently shown to contain only one P450, CYP116B234.

Subsequent mass spectrometric analysis of the P450‐containing fractions following in‐solution tryptic digestion identified CYP116B234 with 63% sequence coverage and a 95% confidence interval. Further analysis of the purified fractions by SDS‐PAGE revealed no distinct single band due to the low expression of CYP116B234; however, a gel slice corresponding to the expected molecular mass of CYP116B234 (~85 kDa) was excised (data not shown). This gel slice was subjected to mass spectrometric analysis following in‐gel tryptic digestion; this confirmed the presence of CYP116B234 with 52% sequence coverage and a 95% confidence interval.

These results indicate that CYP116B234 participates in the catabolism of 2‐HPA but is not involved in phenylalanine metabolism in *R. globerulus* . Consistent with this, Donoso et al. (2021) previously suggested through transcriptomic analysis that the natural function of OhpA was as a 2‐HPA hydroxylase. Our proteomic analysis provides additional evidence supporting the involvement of another CYP116B family enzyme, CYP116B234, in the degradation of 2‐HPA. The origin of 2‐HPA in *R. globerulus* remains unclear, but it does not appear to be a product of the phenylalanine catabolism pathway.

## 4 Conclusion

In this study, a novel self‐sufficient cytochrome P450, CYP116B234, has been isolated and characterised from *R. globerulus* . Previous research has identified three P450 enzymes in *R. globerulus* , namely CYP176A1, CYP108N12 and CYP108N14, involved in the metabolism of monoterpenes such as 1,8‐cineole and *p*‐cymene. In contrast, CYP116B234 demonstrates a distinct functional role, oxidising substituted aromatic compounds such as 2‐HPA and HPPA. CYP116B234 showed a strong substrate preference for 2‐HPA, as reflected in its high NADPH consumption rate and efficient coupling as compared to HPPA as a substrate. Proteomic analysis further suggested that the native function of CYP116B234 is the catabolism of 2‐HPA via the homogentisate pathway. This function parallels that of the *ohpA* gene product (a CYP116B enzyme) in *C. pinatubonensis* JMP134 and the thermophilic CYP116B46 enzyme from *Tepidiphilus thermophilus* (Akter et al. 2024). The complete conservation of all 18 active‐site residues between OhpA and CYP116B234 supports this functional similarity. Interestingly, despite some variations in active‐site residues between CYP116B46 and CYP116B234, their substrate preferences remain consistent. Furthermore, the conservation of active‐site residues between CYP116B5 from *A. radioresistens* S13 and CYP116B234 suggests that CYP116B5's substrate scope could extend beyond the reported alkanes. Given the high similarity of active‐site residues among CYP116B enzymes, further studies are needed to explore the broader substrate scope and fully elucidate the native roles of this enzyme family. Taken together, these findings highlight the metabolic diversity of *R. globerulus* and present CYP116B234 as a valuable addition to the self‐sufficient CYP116 family of enzymes with promising potential for biotechnological applications.

## Author Contributions

**Simran Kundral:** formal analysis, methodology, investigation, validation, writing – original draft. **Hannah Beamish:** methodology, validation, writing – original draft, investigation, formal analysis. **Peter D. Giang:** formal analysis, methodology, investigation, writing – review and editing. **Lauren J. Salisbury:** methodology, investigation, writing – review and editing. **Amanda S. Nouwens:** methodology, investigation, writing – review and editing. **Sunil K. Khare:** investigation, writing – review and editing. **Paul V. Bernhardt:** conceptualization, resources, writing – review and editing. **Jeffrey R. Harmer:** conceptualization, writing – review and editing, resources. **Stephen G. Bell:** writing – review and editing, conceptualization, formal analysis. **James J. De Voss:** resources, conceptualization, writing – review and editing, supervision, formal analysis.

## Conflicts of Interest

The authors declare no conflicts of interest.

## Supporting information


Data S1.

## Data Availability

The data that support the findings of this study are available from the corresponding author upon reasonable request.
