# Supplementary material for: Characterisation of the Self‐Sufficient Cytochrome P450 CYP116B234 From Rhodococcus globerulus and Its Suggested Native Role in 2‐Hydroxyphenylacetic Acid Metabolism
Source: Microb Biotechnol. 2025 Mar 8;18(3):e70125. doi: 10.1111/1751-7915.70125 (PMC11889516; doi:10.1111/1751-7915.70125)
Supplement: Supplementary file 1 — Data S1. [file MBT2-18-e70125-s001.docx]

Supporting Information - Characterisation of the Self-Sufficient Cytochrome P450 CYP116B234 from *Rhodococcus globerulus* and Its Suggested Native Role in 2-Hydroxyphenylacetic Acid Metabolism

*Simran Kundral ^a b c 1^, Hannah Beamish ^b 1^, Peter D. Giang ^b^, Lauren J. Salisbury ^b^, Amanda Nouwens ^b^, Sunil K. Khare ^c d^, Paul V. Bernhardt ^b^, Jeffrey R. Harmer ^e^, Stephen G. Bell ^f^, James J. De Voss  ^b*^*

*^a^ The University of Queensland - Indian Institute of Technology Delhi Research Academy (UQIDRA), India*

*^b^ School of Chemistry and Molecular Biosciences, The University of Queensland, Brisbane 4072, Australia*

*^c^ Enzyme and Microbial Biochemistry Laboratory, Department of Chemistry, Indian Institute of Technology Delhi, India*

*^d^ Department of Biological Sciences, Indian Institute of Science Education and Research Kolkata, India*

*^e^ Centre for Advanced Imaging, Australian Institute for Bioengineering and Nanotechnology, The University of Queensland, Brisbane 4072, Australia*

*^f^ Department of Chemistry, The University of Adelaide, Adelaide, SA, 5005, Australia.*

*** [*j.devoss@uq.edu.au*](mailto:j.devoss@uq.edu.au)

Table of Contents

[Spectroelectrochemistry – inorganic mediator structures 3](#_Toc187669450)

[Amino acid sequence alignment of CYP116B enzymes 4](#_Toc187669451)

[*In silico* model of CYP116B234 7](#_Toc187669452)

[CYP116B234 SDS-PAGE analysis 8](#_Toc187669453)

[CYP116B234 substrate binding analysis 9](#_Toc187669454)

[Redox Characterisation 9](#_Toc187669455)

[*In vitro* catalytic turnover product mass fragmentation profiles 10](#_Toc187669456)

[*In vivo* CYP116B234 catalytic turnovers 11](#_Toc187669457)

[Phenylalanine metabolic pathway 12](#_Toc187669458)

[Proteomic analysis of *R. globerulus* 13](#_Toc187669459)

[Amino-acid sequences 15](#_Toc187669460)

[References 16](#_Toc187669465)

#

# Spectroelectrochemistry – inorganic mediator structures

***
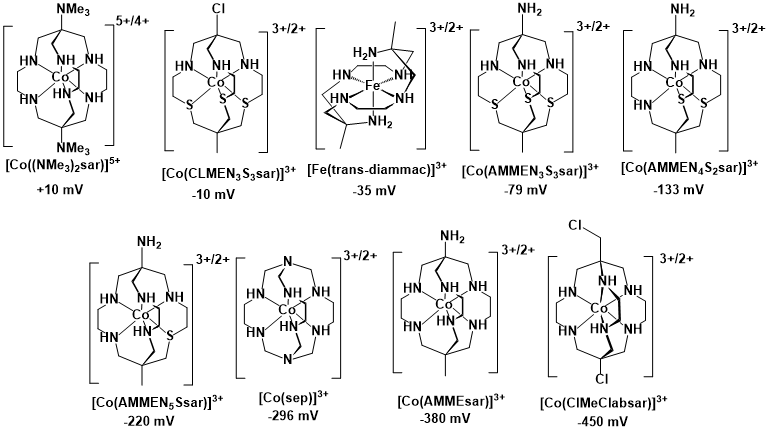
***

Scheme S1: Inorganic redox mediator complexes used in this work and their redox potentials (mV vs. NHE at pH 7) (Bernhardt et al., 2006).

# Amino acid sequence alignment of CYP116B enzymes











Figure S1: The amino acid sequence alignment of characterised class VII enzymes was performed using Clustal Omega web server and visualized by ESpript 3.0 software.

# *In silico* model of CYP116B234


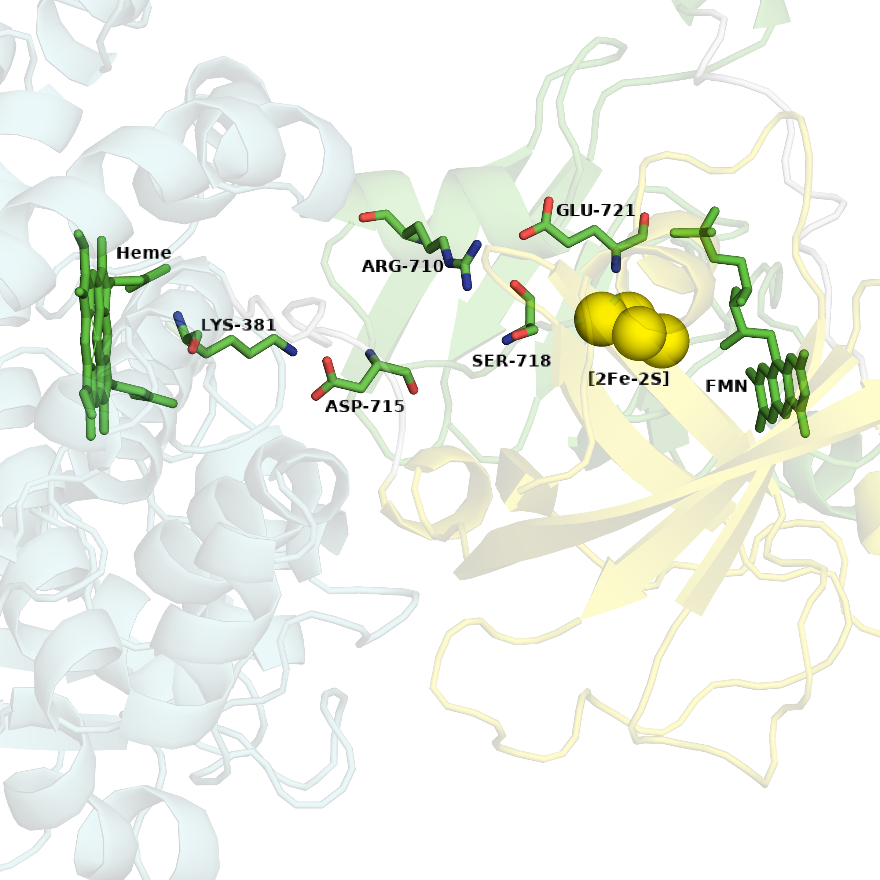


Figure S2: Focused image showing the five key residues involved in the proposed electron shuttling from [2Fe-2S] to heme domain in CYP116B234. The protein structure of CYP116B234 was predicted by AlphaFold 3 program and visualised in PyMOL Molecular Graphics System.

# CYP116B234 SDS-PAGE analysis


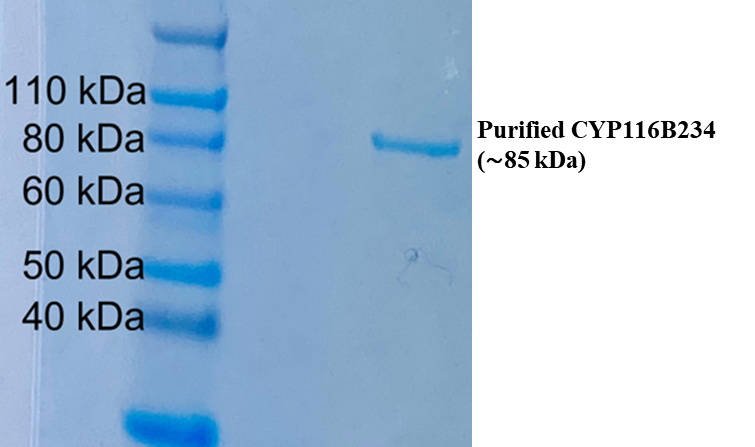


Figure S3: SDS-PAGE (NuPAGE 4 - 12%) analysis of CYP116B234 protein purified using Ni-NTA affinity chromatography. Masses of the protein ladder (ThermoFisher, Novex Sharp Pre-Stained Protein Standard) are indicated.

# CYP116B234 substrate binding analysis

Table S1: Heme spin state shift calculations of substrates tested for binding with CYP116B234 (n = 3) ± standard deviation. n.d represents no spin state shift observed.

| **Compound** | **Spin state shift (%)** |
| --- | --- |
| Phenylacetic acid | 2 ± 1 |
| 3,4-Dihydroxyphenylacetic acid | 5 ± 3 |
| 3-Phenylpropionic acid | 8 ± 5 |
| *trans*-Cinnamic acid | 8 ± 4 |
| α-Pinene | 10 ± 3 |
| 1,8-Cineole | 9 ± 2 |
| (*S*)-α-Terpineol | 5 ± 2 |
| (*S*)-4-Terpineol | 1 ± 1 |
| Decanoic acid | n.d |
| 4-Cholesten-3-one | n.d |

# Redox Characterisation

Figure S4: UV-visible spectra (300 - 800 nm) of spectrochemical titration of **substrate-bound** (100 μM 2-HPA) CYP116B234 (20 µM) showing oxidised (black) and reduced (blue) states.


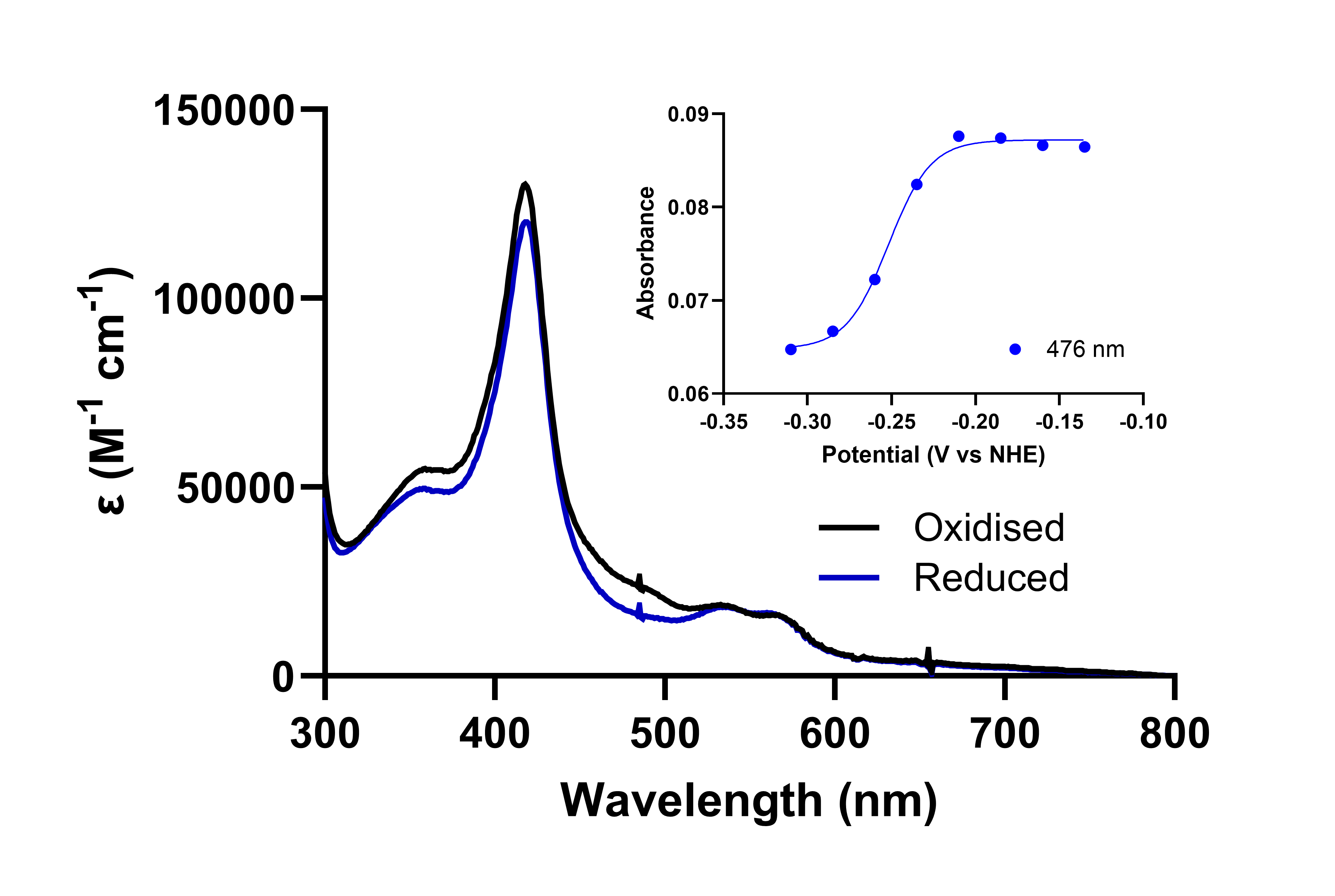


Figure S5: UV-visible spectra (300 - 800 nm) of substrate-free CYP116B234 (20 µM) showing fully oxidised and fully reduced states. Inset selected single wavelength absorption values due to applied potential. The calculated redox potential of the CYP116B234 FMN cofactor was -240 mV vs NHE.

#

# *In vitro* catalytic turnover product mass fragmentation profiles


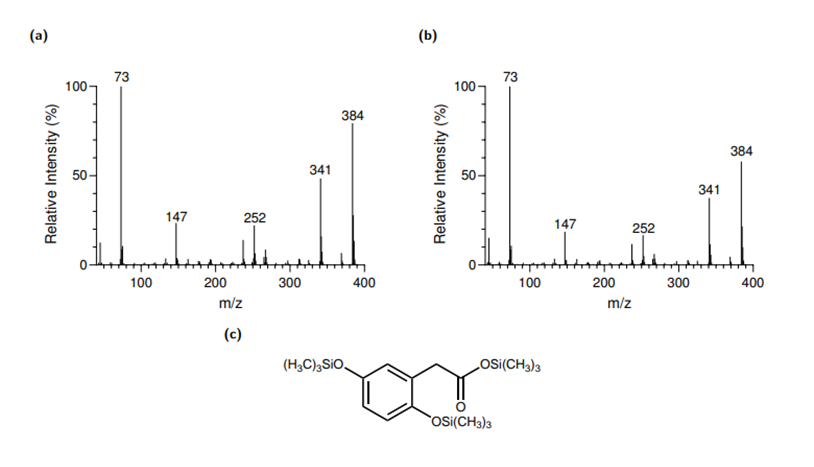


Figure S6: In vitro turnover product of CYP116B234 with 2-hydroxyphenylacetic acid (a) Mass spectrum of BSTFA-TMCS derivatised turnover product with a retention time of 19.4 min (b) Mass spectrum of derivatised homogentisic acid standard with a retention time of 19.4 min and (c) Structure of derivatised homogentisic acid.


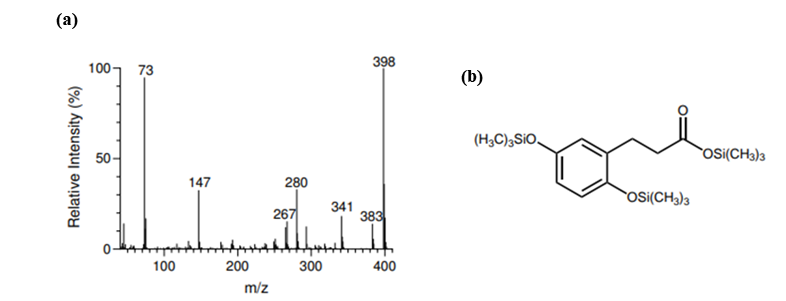

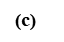

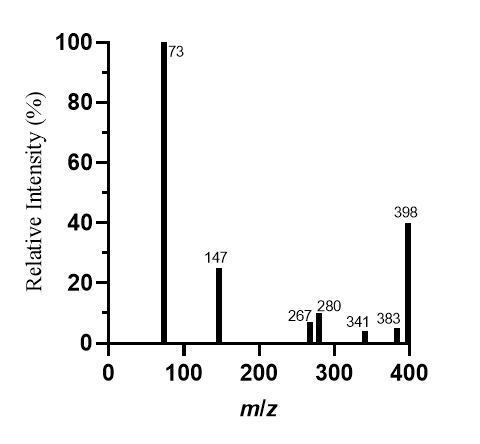


Figure S7: *In vitro* turnover product of CYP116B234 with 3-(2-hydroxyphenyl)propionic acid (a) Mass spectrum of BSTFA-TMCS derivatised turnover product with a retention time of 20.5 min (b) Structure of derivatised 3-(2,5-dihydroxyphenyl)propionic acid and (c) Representative mass fragmentation pattern of TMS-derivatised 3-(2,5-dihydroxyphenyl)propionic acid as reported by Heindl *et al.*, 1985.

*
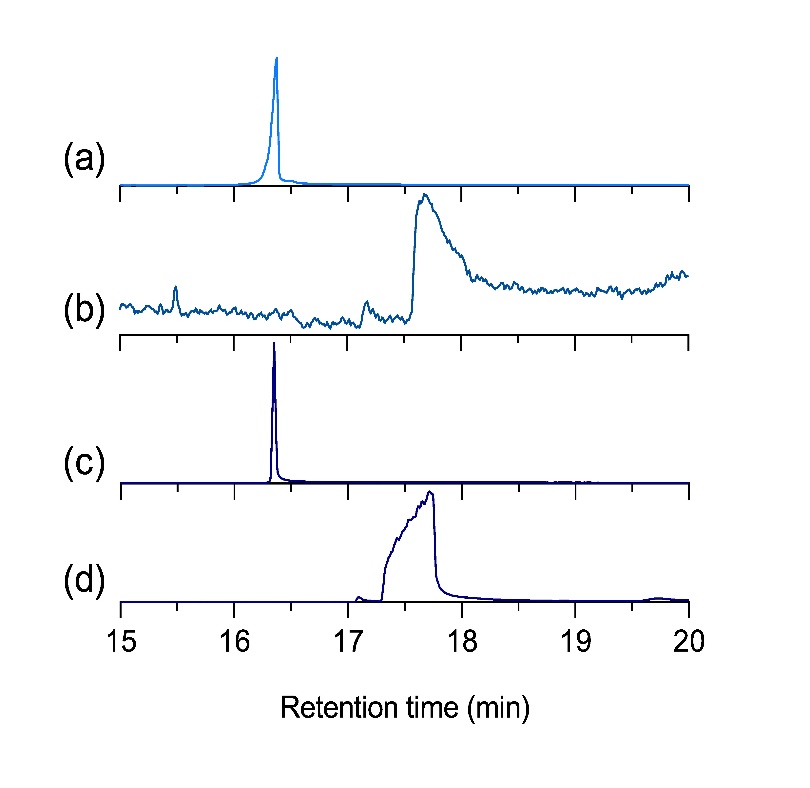
**In vivo* CYP116B234 catalytic turnovers


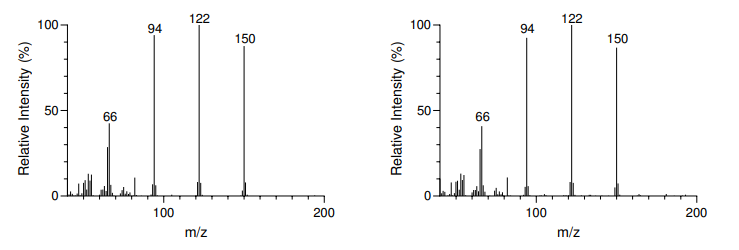


**(a)**

**(b)**

Figure S8: GC-MS traces of in vivo turnovers of 2-hydroxyphenylacetic acid (2-HPA) with CYP116B234 (a) single peak of 2-HPA in negative control (E. coli transformed with an empty vector), (b) 2-HPA in vivo turnover producing homogentisic acid, (c) 2-HPA commercial standard with a retention time of 16.3 min (m/z 296), and (d) homogentisic acid standard with a retention time of 17.6 min (m/z 168). Sample (a) and (c) were derivatised with BSTFA-TMCS before GC-MS analysis.

Figure S9: (a) Mass spectrum of in vivo turnover product of 2-hydroxyphenylacetic acid by CYP116B234, with a retention time of 17.7 min (m/z 168), and (b) Mass spectrum of the homogentisic acid standard, with a retention time of 17.6 min.


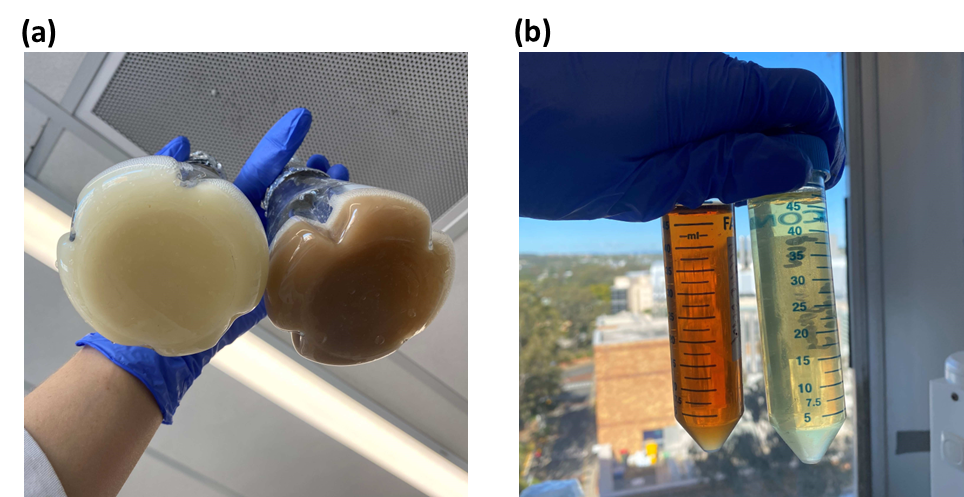


Figure S10: CYP116B234 in vivo 2-hydroxyphenylacetic acid (2-HPA) turnovers (a) E. coli cultures following incubation with 2-HPA. Left flask: negative control (E. coli transformed with an empty vector), Right flask: E. coli expressing CYP116B234 and (b) Cell-free extracts of E. coli cultures following incubation with 2-HPA. Left: E. coli expressing CYP116B234, Right: negative control (E. coli transformed with an empty vector).

# Phenylalanine metabolic pathway

Figure S11: Pathway for the catabolism of Phenylalanine adapted from Arias-Barrau et al., 2004. The intermediates of the catabolic pathway are indicated. Initial steps of the pathway include enzymes (a) PhhA (phenylalanine hydroxylase), (b) PhhB (carbinolamine dehydratase), (c) TyrB (tyrosine aminotransferase), and (d) Hpd (4-hydroxyphenylpyruvate dioxygenase). The homogentisate central pathway is highlighted here. HmgA (homogentisate 1,2-dioxygenase), HmgB (fumarylacetoacetate hydrolase), and HmgC (maleylacetoacetate isomerase).

# Proteomic analysis of *R. globerulus*

After tryptic digestion, peptides were separated using reversed-phase chromatography on a Waters M-Class UPLC system. Samples were loaded onto a Waters NanoEase HSS T3 column (100 Å, 1.8 µm,  300 µm x 150 mm) and chromatography performed at 5 µL/min with column set at 40℃. Mobile phase was held at 3% B for 1 min, followed by a linear gradient from 3 – 45% B over 45 minutes, 45-97% B over 6 min and held at 97% B for 4 min followed by re-equilibration for 4 min. Mobile phase A = 0.1% formic acid in water and B = 0.1% formic acid in AcN. Eluted peptides were directly analysed on a ZenoTof 7600 instrument (ABSciex) using an OptiFlow Micro/MicroCal source. Source conditions included curtain gas = 35 psi, CAD gas = 7, Gas 1 = 20 psi, Gas 2 = 15 psi, source temp = 150℃, spray voltage = 5000 V, DP = 80, CE = 10. Peptides were analysed by Data Dependent Analysis with MS TOF scan across 400-1750 m/z performed for 0.2 sec followed by data dependent analysis of up to 20 peptides with intensity greater than 150 counts, across 50-2000 m/z (0.035 sec per spectra) using dynamic collision energy. Zeno pulsing was on, with threshold set to 100000 cps. Former candidate ions were excluded for 6 sec after 1 occurrence.

MS data was processed with ProteinPilot v5.0.2. Search parameters included: sample type = idneitication, Cys alkyation = acrylamide, Digestion = trypsin, Instrument = QSTAR Elite, Search effort = Thorough, FDR analysis = yes, and using a custom database of all *Rhodococcus* entries listed on Uniprot (April, 2021) and the sequence of CYP116B234, combined with the cRAP database (<ftp://ftp.thegpm.org/fasta/cRAP>).

Table S2: SWATH- MS analysis of cell lysate of *R. globerulus* grown on Phenylalanine. The table lists enzymes induced in the presence of phenylalanine, along with their annotated names, genome IDs, sequence coverage (%Cov at 95% confidence), UniProt accession numbers (with % amino acid identity to the observed enzymes), and associated organisms.

| **Enzyme observed (annotated names); genome IDs** | **%Cov(95)** | **Uniprot accession no. (% amino acid identity to observed enzyme); organism** |
| --- | --- | --- |
| Phenylalanine-4-hydroxylase (PhhA) | 72 | P43334 (85.7%);  *Pseudomonas aeruginosa* ATCC 15692 |
| Putative pterin-4-alpha-carbinolamine dehydratase (PhhB) | 100 | Q3KG65 (96.6%);  *Pseudomonas fluorescens* Pf0-1 |
| Aromatic-amino-acid aminotransferase (TyrB) | 64 | O85746 (71.5%);  *Klebsiella pneumoniae* |
| 4-hydroxyphenylpyruvate dioxygenase (Hpd) | 95 | P80064 (89.1%);  *Pseudomonas* sp. P.J. 874 |
| Homogentisate 1,2-dioxygenase (HmgA)^a^ | 85 | A5W8Z1 (86.8%);  *Pseudomonas putida* ATCC 700007 |
| Ureidoglycolate lyase (HmgB) | 59 | Q88E48* (87.8%);  *Pseudomonas putida* ATCC 47054 |

*UniProtKB unreviewed (TrEMBL)

Table S3: SWATH- MS analysis of cell lysate of *R. globerulus* grown on 2-hydroxyphenylacetic acid. The table lists enzymes induced in the presence of 2-hydroxyphenylacetic acid, along with their annotated names, genome IDs, sequence coverage (%Cov at 95% confidence), UniProt accession numbers (with % amino acid identity to the observed enzymes), and associated organisms.

| **Enzyme observed; genome IDs** | **%Cov(95)** | **Uniprot accession no. (% amino acid identity to observed enzyme); organism** |
| --- | --- | --- |
| Cytochrome P450 (CYP116B234) | 8 | Q46QG0 (65%);  *Cupriavidus pinatubonensis* JMP 134 |
| Homogentisate 1,2-dioxygenase (HmgA)^a^ | 60 | A5W8Z1 (86.8%);  *Pseudomonas putida* ATCC 700007 |
| Predicted Homogentisate 1,2-dioxygenase^b^ (HmgA) | 77 | A0A0D8HTK9* (99%);  *Rhodococcus* sp. AD45 |
| Ureidoglycolate lyase (HmgB) | 38 | Q88E48* (87.8%);  *Pseudomonas putida* ATCC 47054 |

*UniProtKB unreviewed (TrEMBL)

# Amino-acid sequences

# **CYP116B234**

# MSEVMNGCPIDHAALNSGCPVSGKAASFNPFGGDYQVDPAASLRWSRDEEPVFFSPEIGYWVVTRYSDVKAVFRDNILFSPSIALEKITPVSEEATATLAKYDYAMSRTLVNEDEPAHMPRRRALMDPFTPAELVHHEPMVRRLVREYVDRFVNSGKVDLVDEMLWEVPLTVALHFLGVPEEDMDDLRKYSIAHTVNTWGRPAPEEQVAVAEAVGKFWQYAGTVLEKMRKDPSGHGWMPYGLRVQKEQPEVVTDSYLHSMMMAGIVAAHETTANASANAFRLLLENRSVWQEICEDPSLIPNAVEECLRHSGSVAAWRRLATADTRIGDIDIPKGSKLLIVTSSANRDDRHFESGDEFDIRRENSSDHLTFGYGSHQCMGKNLARMEMQIFLEEITTRLPHLELVPDQEFTYLPNTSFRGPDHVWVQWNPDANPELRDASILLRRQPVKVGEPSKTNISRTVRVEAITPAADGVVTVTLSDPSGKALPKWTPGAHIDIDLGDLTRQYSLCGNPHDLSRYEIAVREESESRGGSRYVHHTLEAGNTLKMRGPRNHFKLDPAAERYVFVAGGIGITPIVTMADHAKAAGKDYEIHYCGRDVATMAMLDRLNADHSDHLVVHSSALGNRLDIATVFSTATAHTQIYSCGPERLLSALEEATAHWPEDSLHVEHFTSTLATLDPSNEHAFEVELRDSGLTIQVAADQTVLDALRASNIDIPSDCEEGLCGSCEAPVLDGEVDHRDMVLTKTERAQNKSMMTCCSRACGQKITLAL

# **Phenylalanine-4-hydroxylase (Incomplete sequence)**

# EEELDYLQEPDIFHEIFGHCPLLTNPWFAEFTHTYGKLGLQATKEERVYLARLYWMTIEFGLVDTPQGRKIYGGGILSSPKETVYSLSDAPEHQAFDPMEAMRTPYRIDILQPLYFALPNLKRLFDLAHEDIMGMVHTAMKMGLHAPKFPPKVAA

**Putative pterin-4-alpha-carbinolamine dehydratase**

MNALNQAHCEACRADAPQVSDEELPVLIKQIPDWNIEVRDGIMQLEKVFLFKNFKHALAFTNAVGEISEAEGHHPGLLTEWGKVTVTWWSHSIKGLHRNDFIMAARTDEVAKTAEGRK

**Aromatic-amino-acid aminotransferase**

MFKHVDAYAGDPILSLMETFKADPRANKVNLSIGLYYDAAGVVPQLAAVGEAEKRMAGQPHEASLYLPMEGLSAYRQAIQALLFGADHPAVQGGRVATVQTVGGSGALKVGADFLKRYFPESQVWVSNPTWDNHRAIFEGAGFKVNTYPYFDQGTRGLDFDGMLSTLQGLPANSIVLLHPCCHNPTGVDLSQAQWQQVIEVVKARNLIPFLDIAYQGFGEGLVEDAYAIREAARAGVPCLVSNSFSKIFSLYGERVGGLSVVCDDADTAQSVLGQLKATVRRNYSSPPNFGAQLVAAVLGDAVLNAQWAAEVEQMRLRILDMRQGLVDALAVLLPGQDFQFFLSQRGMFSYTGLSVEQVRRLRDEFGVYLIDSGRVCMSGLRPDNLQQVAEAIAAVQA

**4-hydroxyphenylpyruvate dioxygenase**

MADIFENPMGLMGFEFIEFASPTPGVLEPIFQIMGFTKVATHRSKDVHLYRQGDINLILNNEPKSIASYFAAEHGPSVCGMAFRVRNAHEAYARALELGAQPVEIETGPMELRLPAIKGIGGAPLYLIDRYEEGSSIYDIDFNFIEGVDRHPVGAGLKIIDHLTHNVYRGRMAYWAAFYEKLFNFREIRYFDIKGEYTGLTSKAMTAPDGMIRIPLNEESSKGAGQIEEFLMQFNGEGIQHVAFLTDDLLKSWDALKKLGMRFMTAPPQTYYEMLEGRLPGHGEPVDQLQARGILLDGSSQPDDKRLLLQIFSETLMGPVFFEFIQRKGDDGFGEGNFKALFESIERDQVRRGVLNAE

**Homogentisate 1,2-dioxygenase^a^**

MNLDSTPVLDYLSGFGNEFASEALPGALPVGQNSPQKAPYGLYAELFSGTAFTMTRSEMRRTWLYRIRPSALHPRFERLERQLAGGPLGAVTPNRLRWSPQAIPSEPTDFIDGWVAMAANSGSEKPAGISIYTYCANRSMERVFFNADGELLLVPELGRLRLVTELGVLVVEPLEIAVVPRGLKFRVELLDSQARGYLAENHGAPLRIPDLGPIGSNGLANPRDFLSPVAHYEEHQGPVQLVQKFLGELWGCELNHSPLDVVAWHGNNVPYKYDLRRFNTIGTVSFDPPDPAIFTVLTSPTSVPGMANLDFVIFPPRWMVAENTFRPPWFHRNLMNEFMGLIKGEYDAKAEGFLPGGASLHSCMSAHGPDAETCAKAIAVELAPNKIDNTMAFMFETSQVLRPSRHALECPQLQADYDSCWASLPSTFTPNRR

**Ureidoglycolate lyase**

MLAAGLFDGKARAAVEATRGSDLNAFFALGRVARVALRERLLELLGEHSEHQAALKPLLLASGECQLHLPAQIGDYTDFYVGIEHAKNVGKLFRPDNPLLPNYKYVPIGYHGRASTIRPSGTDVRRPKGQTLPAGQSEPSFGPCARLDYELELGIWIGQGNEMGESIPVAEAAEHIAGFCLLNDWSARDIQAWEYQPLGPFLSKSFISTVSPWVVTAEALEPFRCAQPSRPEGDPQPLSYLLDKRDQAGGAFDIELEVLLLTERMREQNLPAHRLTLSNTLSMYWTVAQMVAHHSVNGCQLQPGDLFGSGTLSGAQPGQFGSLLEITQGGKEPVQLASGEVRKFLEDGDEIILRARCVADGVASIGFGECRGKILPAH

**Homogentisate 1,2-dioxygenase^b^**

MAFYRQLGNVPPKRHTQHRDEAGNLYYEELMGEEGFSSDSSLLYHREIPSAIVDATLWELRDQSTTPNHPLKPRHLKLHDLFPESIRADTDVVTGRRLILGNADVRLSYVIAGAASPLYRNAIGDEMVYIESGEATVETVFGALEAREGDYVLIPMSTTHRWIPKGDKPLRAYAIEANSHIVPPKRYLSRFGQLLENAPFCERDLHGPTAPLIAEGTDVEVLVKHRTSAGIVGTRMVYPTHPFDVVGWDGCLYPYTFNISDYEPITGRVHQPPPAHQAFEGNNFVICNFVPRKVDYHPLAIPVPYYHSNVDSDEIMFYCGGDYEARKGSGIGQGSISVHPGGHAHGPQPGAYERSIGAEFFDELAVMVDTFRPLELGEGALACEDDNYAWSWAGRGPAQ

# References

1. Arias-Barrau, E., Olivera, E.R., Luengo, J.M., Fernández, C., Galán, B., García, J.L., et al. (2004) The Homogentisate Pathway: a Central Catabolic Pathway Involved in the Degradation of l-Phenylalanine, l-Tyrosine, and 3-Hydroxyphenylacetate in *Pseudomonas putida*. *J Bacteriol* **186**: 5062–5077.
2. Bernhardt, P.V., Chen, K.-I., and Sharpe, P.C. (2006) Transition metal complexes as mediator-titrants in protein redox potentiometry. *JBIC J Biol Inorg Chem* **11**: 930–936.
3. Heindl, A., Rau, O., and Spiteller, G. (1985) Identification of aromatic dihydroxy acids in biological fluids. *Biol Mass Spectrom* **12**: 59–66.
